# Darwin wasps (Hymenoptera, Ichneumonidae) of the Nature Reserve of Pantalica (Sicily, Italy)

**DOI:** 10.3897/BDJ.13.e175760

**Published:** 2025-12-18

**Authors:** Filippo Di Giovanni, Davide Badano, Davide Dal Pos, Claudio Cucini, Rebecca Funari, Erich Diller, Oleksandr Varga, Stefano Cantone, Andrea Di Giulio

**Affiliations:** 1 University of Siena, Siena, Italy University of Siena Siena Italy; 2 National Biodiversity Future Center, Palermo, Italy National Biodiversity Future Center Palermo Italy; 3 University of Central Florida, Orlando, United States of America University of Central Florida Orlando United States of America; 4 Zoologische Staatssammlung München, Munich, Germany Zoologische Staatssammlung München Munich Germany; 5 I.I. Schmalhausen Institute of Zoology of National Academy of Sciences of Ukraine, Kyiv, Ukraine I.I. Schmalhausen Institute of Zoology of National Academy of Sciences of Ukraine Kyiv Ukraine; 6 University of Roma Tre, Rome, Italy University of Roma Tre Rome Italy

**Keywords:** biodiversity, checklist, Ichneumoninae, parasitoids, Phaeogenini, Sicily

## Abstract

**Background:**

The hymenopteran family Ichneumonidae (also known as Darwin wasps) represents one of the largest families of parasitoid insects. However, faunistic research is needed to fill the gaps in the knowledge of the biodiversity of this group in the Mediterranean area and Italy.

**New information:**

A faunistic survey on Darwin's wasps of the Nature Reserve of Pantalica (Siracusa, Sicily, Italy) is presented. A total of 81 species of ichneumonids belonging to 19 subfamilies were identified. Of these, 42 species are new records for Sicily and nine species are new records for Italy. The subfamilies Adelognathinae and Oxytorinae are reported for the first time in Sicily.

*Misetus obscurus* Berthoumieu, 1897 **stat. rev.** (Ichneumoninae, Phaeogenini), originally described as a colour form of *M. oculatus* Wesmael, 1845, is raised to species level, based on both morphological and molecular data.

## Introduction

With over 25,000 known species and an estimated diversity ranging between 60,000 and 100,000 species (Meier et al. 2024), Ichneumonidae (commonly known as Darwin wasps) represent one of the most diverse insect groups (Yu et al. 2016). The Italian fauna of this group comprises more than 2,260 species, although many of the records are fragmented and mostly concentrated in the northern regions of the peninsula (Di Giovanni et al. 2015, Di Giovanni et al. 2025a).

In the most recent checklist of the Italian fauna, Scaramozzino (1995) reports approximately 175 species for Sicily. However, this list lacks several records, many of which are found in little-known or very old papers, such as those by De Stefani Perez (e.g. De Stefani (1895)). In addition to these records, around 140 species have been added to the list since the 1995s' checklist, largely due to some major faunistic contributions (e.g. Pagliano and Scaramozzino (1995), Pagliano (2003), Turrisi et al. (2007), Pagliano (2009), Riedel and Turrisi (2013), Zwakhals and Turrisi (2014)). To date, by integrating historical records, unreported data and new findings, the Darwin wasp fauna of the island comprises about 450 species (Di Giovanni & Dal Pos, unpublished data).

Recent studies have shown (e.g. Di Giovanni et al. (2015), Di Giovanni et al. (2025a), Di Giovanni et al. (2025b)) that a comprehensive inventory of Ichneumonidae in the Italian fauna is still far from complete. In particular, for many of the twenty administrative regions into which Italy is divided, data are scarce or outdated and much of the diversity of the group in the southernmost part of the peninsula and in the major islands (Sardinia, Sicily) remains largely unexplored.

## Materials and methods

The records presented in this study derive from a sampling campaign conducted in the Nature Reserve of Pantalica (Syracuse, Italy)(GPS coordinates 37°07'46.95"N, 15°01'10.70"E; altitude 236-244 m a.s.l) This protected area, located between the Anapo and Calcinara Rivers, is of exceptional geological, archaeological and faunal significance. The sampling sites are situated in habitats resembling Mediterranean shrubland, with *Quercus ilex*. The evergreen, thermo-Mediterranean species form a structurally complex vegetation layer that creates shaded microhabitats and contributes to relatively stable microclimatic conditions. A total of eight sampling events were carried out, using both Malaise and light traps, between 27 April and 21 August 2023, with closer intervals in spring and more widely spaced intervals in summer (sampling dates: 27/04; 05/05; 17/05; 27/05; 17/06; 09/07; 12/08; 21/08). The research is part of a broader project on the arthropod diversity in the Reserve, by the Entomology and Parasitology Lab., Department of Science, Roma Tre University (Rome, Italy).

The species are listed in alphabetical order, by subfamily, genus and species. For each species, the general distribution, occurrence in Italy and notes on morphology are provided. General distribution data follows Taxapad (Yu et al. 2016), supplemented by more recent taxonomic revisions. For the distribution in Italy, only the main bibliographic references are cited, to complement the data from the most recent available checklist (Scaramozzino 1995). The subdivision of Italian territory into North Italy, South Italy, Sicily and Sardinia follows Minelli et al. (1995); "North Italy" and "South Italy" should, therefore, be considered proper nouns (see also Di Giovanni et al. (2025a)).

All collected specimens are preserved in the personal collection of the first author (FDGC, Filippo Di Giovanni Collection, Siena, Italy).

Photographs were taken with a Nikon D5300 digital camera attached to a Leica Z16 APO stereoscope. Images were acquired using StackShoot TM multiple-focus imaging system and stacked in a single in-focus image using Zerene Stacker software (version 1.04). Images of *Orthocentrus protervus* were taken with a Leica Z16 APO microscope, equipped with Leica FLEXACAM C1 camera and processed by LAS Core software.

For *Misetus obscurus*, DNA was extracted using the Wizard® SV Genomic DNA Purification System (Promega, US), following the manufacturer’s protocol. Amplification of the mitochondrial DNA fragment Cytochrome Oxidase subunit I (COX1) was performed as described in Badano et al. (2024) using the forward primer LCO1490 and the reverse primer HCO2198 (Folmer et al. 1994). Sequencing was performed at BMR Genomics (Padua, Italy) using the same primers and the Sanger sequencing method on an automated DNA sequencer. Resulting electropherograms were manually inspected with Sequencher 5.4.6 (Gene Codes, US), species identification being achieved with a BLAST strategy through the BOLD System V5 and the NCBI databases. P-distances were calculated in R v.4.5.0 (Team 2025) using the *ape* package (Paradis and Schliep 2018) with the function dist.dna (model = "raw", pairwise.deletion = TRUE).

## Checklists

### Checklist of Darwin waps (Ichneumonidae) of the Nature Reserve of Pantalica (Siracusa, Italy)

#### Adelognathinae

Thomson, 1888

5341270A-51DC-5648-AF7E-3FB5D98B4604

#### Adelognathus

Holmgren, 1857

5AC6B4C4-4ADD-5689-832C-D964F5732908

#### Adelognathus punctulatus

Thomson, 1883

6CEA0F40-43CD-55E4-861B-82073ABB0D5B

##### Materials

- **Type status:** Other material. **Occurrence:** recordedBy: S. Cantone, A. Di Giulio; individualCount: 1; sex: female; occurrenceID: F5F639C2-7FEE-59ED-9CA1-201023DBBC8F; **Location:** country: Italy; countryCode: IT; stateProvince: Sicily; municipality: Sortino, Siracusa; locality: Riserva Naturale di Pantalica; **Identification:** identifiedBy: F. Di Giovanni; dateIdentified: 2025; **Event:** eventDate: 09/07/2023; year: 2023; month: 7; day: 9; **Record Level:** institutionCode: FDGC
- **Type status:** Other material. **Occurrence:** recordedBy: S. Cantone, A. Di Giulio; individualCount: 2; sex: females; occurrenceID: 2FDE0A5C-652B-58EA-A393-3CE580D2D267; **Location:** country: Italy; countryCode: IT; stateProvince: Sicily; municipality: Sortino, Siracusa; locality: Riserva Naturale di Pantalica; **Identification:** identifiedBy: F. Di Giovanni; dateIdentified: 2025; **Event:** eventDate: 12/08/2023; year: 2023; month: 8; day: 12; **Record Level:** institutionCode: FDGC

##### Distribution

Nearctic; Palaearctic (Eastern Palaearctic; Western Palaearctic).

##### Notes

Species recorded for North Italy (Kasparyan 1990, Scaramozzino 1995, Pagliano 2009). This is the first record of the subfamily Adelognathinae and of the species for Sicily.

#### Anomaloninae

Viereck, 1918

ECB82079-FD3B-5CEF-893D-A7CC153BF17F

#### Anomalonini

Viereck, 1918

35510E67-712B-531B-A981-31B84A4F8E9C

#### Anomalon

Panzer, 1804

BC0A9FB1-D9A6-5051-B5C2-A580D829891F

#### Anomalon cruentatum

(Geoffroy, 1785)

15E4D209-7A45-5996-8D69-E720D732CA75

##### Materials

- **Type status:** Other material. **Occurrence:** recordedBy: S. Cantone, A. Di Giulio; individualCount: 1; sex: male; occurrenceID: 8DED9C7C-6D3B-509B-B0BF-4036F86146D5; **Location:** country: Italy; countryCode: IT; stateProvince: Sicily; municipality: Sortino, Siracusa; locality: Riserva Naturale di Pantalica; **Identification:** identifiedBy: F. Di Giovanni; dateIdentified: 2025; **Event:** eventDate: 09/07/2023; year: 2023; month: 7; day: 2; **Record Level:** institutionCode: FDGC

##### Distribution

Indomalyan; Palaearctic (Eastern Palaearctic; Western Palaearctic).

##### Notes

Species present throughout Italy, including the major islands (Scaramozzino 1995).

#### Gravenhorstiini

Enderlein, 1912

4FC4F30E-B5BE-5D36-BCEB-99F052062FC7

#### Agrypon

Förster, 1860

07687B64-FB48-5660-82C1-36249EA20BC4

#### Agrypon delarvatum

(Gravenhorst, 1829)

2C2015D5-1735-5FDF-ACDE-5FB3348C7955

##### Materials

- **Type status:** Other material. **Occurrence:** recordedBy: S. Cantone, A. Di Giulio; individualCount: 1; sex: female; occurrenceID: 70081EDF-C411-5ACE-A559-01AF6EC67F61; **Location:** country: Italy; countryCode: IT; stateProvince: Sicily; municipality: Sortino, Siracusa; locality: Riserva Naturale di Pantalica; **Identification:** identifiedBy: F. Di Giovanni; dateIdentified: 2025; **Event:** eventDate: 17/06/2023; year: 2023; month: 6; day: 17; **Record Level:** institutionCode: FDGC

##### Distribution

Palaearctic (Eastern Palaearctic; Western Palaearctic).

##### Notes

Species recorded for North and South Italy (Faggioli 1933, Scaramozzino 1995, Pagliano 2009). This is the first record of the species for Sicily.

#### Banchinae

Wesmael, 1845

B6686BF2-BECC-56EF-810A-67DB4BD4CD6A

#### Atrophini

Seyrig, 1932

A088563E-09C8-51A7-84C1-9F12D89BFD9A

#### Lissonota

Gravehorst, 1829

9C1EA548-3503-5359-9BFD-134AFF8B78FB

#### Lissonota carbunculus

Johansson, 2024

63C9F038-A4DF-506B-9E8E-2A504CB33355

##### Materials

- **Type status:** Other material. **Occurrence:** recordedBy: S. Cantone, A. Di Giulio; individualCount: 23; sex: 3 females, 20 males; occurrenceID: 22FFCB71-F751-52B8-A5BF-7C7AE9A6CB2D; **Location:** country: Italy; countryCode: IT; stateProvince: Sicily; municipality: Sortino, Siracusa; locality: Riserva Naturale di Pantalica; **Identification:** identifiedBy: F. Di Giovanni & N. Johansson; dateIdentified: 2025; **Event:** eventDate: 17/05/2023; year: 2023; month: 5; day: 17; **Record Level:** institutionCode: FDGC
- **Type status:** Other material. **Occurrence:** recordedBy: S. Cantone, A. Di Giulio; individualCount: 2; sex: 2 females; occurrenceID: 638D6D50-5A81-5249-93F1-705D807BD386; **Location:** country: Italy; countryCode: IT; stateProvince: Sicily; municipality: Sortino, Siracusa; locality: Riserva Naturale di Pantalica; **Identification:** identifiedBy: F. Di Giovanni & N. Johansson; dateIdentified: 2025; **Event:** eventDate: 17/06/2023; year: 2023; month: 6; day: 17; **Record Level:** institutionCode: FDGC

##### Distribution

Palaearctic (Western Palaearctic) (Johansson 2024).

##### Notes

Species described from France and Sardinia (Johansson 2024). This is the first record of the species for Sicily.

##### Diagnosis

Compared to the original description of the species (Johansson 2024), the specimens examined show the following differences: the small central reddish spot on mesoscutum, as well as the reddish-yellow shoulder marks in males, sometimes lacking; the red margin on metasomal tergites III-IV in females more extensive, with sometimes metasomal tergite IV almost entirely red, except for black spots at the base laterally; meso- and metapleuron in males sometimes almost entirely black.

#### Lissonota corsicator

Aubert, 1972

32CF9368-1E1D-5B0F-88FC-9AF186143ECD

##### Materials

- **Type status:** Other material. **Occurrence:** recordedBy: S. Cantone, A. Di Giulio; individualCount: 1; sex: female; occurrenceID: 37C96BBA-9C13-50B3-9BFE-B3C32D95B921; **Location:** country: Italy; countryCode: IT; stateProvince: Sicily; municipality: Sortino, Siracusa; locality: Riserva Naturale di Pantalica; **Identification:** identifiedBy: F. Di Giovanni & N. Johansson; dateIdentified: 2025; **Event:** eventDate: 27/04/2023; year: 2023; month: 4; day: 27; **Record Level:** institutionCode: FDGC

##### Distribution

Palaearctic (Western Palaearctic) (Johansson 2024).

##### Notes

Species recorded for North Italy, as a subspecies of *Lissonota bicincta* Szépligeti, 1899 (Scaramozzino 1995). This is the first record of the species for Sicily.

##### Diagnosis

With respect to the re-description of the species in Johansson (2024), the specimen collected in Sicily is slightly darker, with the hind corner of pronotum black and with the posterior margin on metasomal tergite II narrowly reddish.

#### Lissonota fuscator

Johansson, 2024

5BC7F59F-4D92-5845-BF4D-EEFC3DBD3B07

##### Materials

- **Type status:** Other material. **Occurrence:** recordedBy: S. Cantone, A. Di Giulio; individualCount: 1; sex: female; occurrenceID: ED8F82F0-C3A5-5682-8BBF-F7CF5BFDFA36; **Location:** country: Italy; countryCode: IT; stateProvince: Sicily; municipality: Sortino, Siracusa; locality: Riserva Naturale di Pantalica; **Identification:** identifiedBy: F. Di Giovanni & N. Johansson; dateIdentified: 2025; **Event:** eventDate: 27/04/2023; year: 2023; month: 4; day: 27; **Record Level:** institutionCode: FDGC

##### Distribution

Palaearctic (Western Palaearctic) (Johansson 2024).

##### Notes

Species described from France, Great Britain and North Italy (Johansson 2024). This is the first record of the species for Sicily.

##### Diagnosis

Compared to the original description (Johansson 2024), the specimen collected in Sicily has the mid- and hind trochantelli almost entirely reddish-yellow and the hind tibia externally sub-basally and apically reddish-brown.

#### Lissonota pleuralis

Brischke, 1880

1001E7FC-76BC-5B4D-8C4F-C5AB7BF110AF

##### Materials

- **Type status:** Other material. **Occurrence:** recordedBy: S. Cantone, A. Di Giulio; individualCount: 3; sex: females; occurrenceID: A1554B4D-21AC-5178-B8D7-0070D894CD97; **Location:** country: Italy; countryCode: IT; stateProvince: Sicily; municipality: Sortino, Siracusa; locality: Riserva Naturale di Pantalica; **Identification:** identifiedBy: F. Di Giovanni & N. Johansson; dateIdentified: 2025; **Event:** eventDate: 17/05/2023; year: 2023; month: 5; day: 17; **Record Level:** institutionCode: FDGC
- **Type status:** Other material. **Occurrence:** recordedBy: S. Cantone, A. Di Giulio; individualCount: 2; sex: females; occurrenceID: BFA1B58E-DF92-5CE9-821E-8BE06F14E147; **Location:** country: Italy; countryCode: IT; stateProvince: Sicily; municipality: Sortino, Siracusa; locality: Riserva Naturale di Pantalica; **Identification:** identifiedBy: F. Di Giovanni & N. Johansson; dateIdentified: 2025; **Event:** eventDate: 17/06/2023; year: 2023; month: 6; day: 17; **Record Level:** institutionCode: FDGC

##### Distribution

Palaearctic (Eastern Palaearctic; Western Palaearctic) (Johansson 2024).

##### Notes

**Figure 1.**
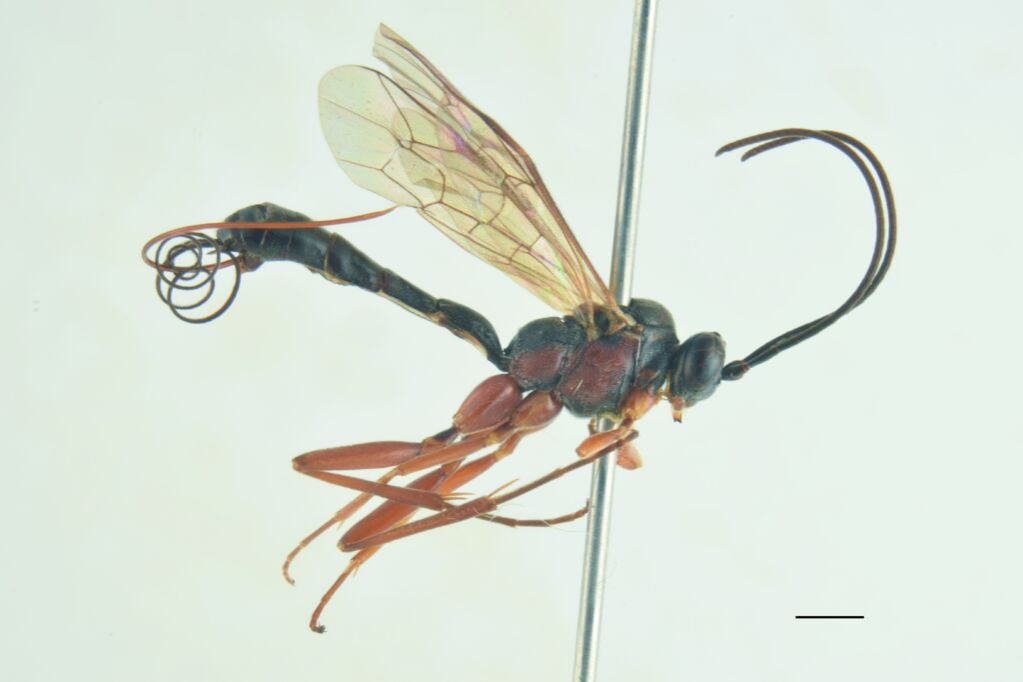
*Lissonota pleuralis* Brischke, 1880, female, habitus. Scale bar 1 mm.

This is the first record of the species for Italy (Fig. 1).

#### Banchini

Wesmael, 1845

49577064-59D9-5EA4-804E-E482F5DD397D

#### Exetastes

Gravenhorst, 1829

C1530C0B-C999-585C-BFC7-3A8E16BE7F56

#### Exetastes adpressorius

(Thunberg, 1822)

D4C753D0-8464-5811-9EBE-46B3F7D623CB

##### Materials

- **Type status:** Other material. **Occurrence:** recordedBy: S. Cantone, A. Di Giulio; individualCount: 2; sex: females; occurrenceID: AF2E4220-91C7-5D62-8A01-3FAE75B2A3A2; **Location:** country: Italy; countryCode: IT; stateProvince: Sicily; municipality: Sortino, Siracusa; locality: Riserva Naturale di Pantalica; **Identification:** identifiedBy: F. Di Giovanni; dateIdentified: 2025; **Event:** eventDate: 27/04/2023; year: 2023; month: 4; day: 27; **Record Level:** institutionCode: FDGC
- **Type status:** Other material. **Occurrence:** recordedBy: S. Cantone, A. Di Giulio; individualCount: 2; sex: females; occurrenceID: 87B295D4-6949-5BC7-BBE2-E5C317185AF5; **Location:** country: Italy; countryCode: IT; stateProvince: Sicily; municipality: Sortino, Siracusa; locality: Riserva Naturale di Pantalica; **Identification:** identifiedBy: F. Di Giovanni; dateIdentified: 2025; **Event:** eventDate: 05/05/2023; year: 2023; month: 5; day: 5; **Record Level:** institutionCode: FDGC
- **Type status:** Other material. **Occurrence:** recordedBy: S. Cantone, A. Di Giulio; individualCount: 2; sex: females; occurrenceID: 12C9E63B-3E14-5BBD-B3E9-5B6A34228576; **Location:** country: Italy; countryCode: IT; stateProvince: Sicily; municipality: Sortino, Siracusa; locality: Riserva Naturale di Pantalica; **Identification:** identifiedBy: F. Di Giovanni; dateIdentified: 2025; **Event:** eventDate: 17/05/2023; year: 2023; month: 5; day: 17; **Record Level:** institutionCode: FDGC
- **Type status:** Other material. **Occurrence:** recordedBy: S. Cantone, A. Di Giulio; individualCount: 1; sex: female; occurrenceID: F6216F7C-0C0C-5EA3-BD49-3F84CB54BF31; **Location:** country: Italy; countryCode: IT; stateProvince: Sicily; municipality: Sortino, Siracusa; locality: Riserva Naturale di Pantalica; **Identification:** identifiedBy: F. Di Giovanni; dateIdentified: 2025; **Event:** eventDate: 17/06/2023; year: 2023; month: 6; day: 17; **Record Level:** institutionCode: FDGC
- **Type status:** Other material. **Occurrence:** recordedBy: S. Cantone, A. Di Giulio; individualCount: 1; sex: female; occurrenceID: 3887B613-5DBD-5E35-81A8-08ECF8CA24D7; **Location:** country: Italy; countryCode: IT; stateProvince: Sicily; municipality: Sortino, Siracusa; locality: Riserva Naturale di Pantalica; **Identification:** identifiedBy: F. Di Giovanni; dateIdentified: 2025; **Event:** eventDate: 09/07/2023; year: 2023; month: 7; day: 9; **Record Level:** institutionCode: FDGC

##### Distribution

Nearctic; Palaearctic (Eastern Palaearctic; Western Palaearctic) (Riedel 2023).

##### Notes

Species present throughout Italy, including the major islands (Scaramozzino 1995).

#### Campopleginae

Förster, 1869

0743B317-B8E1-549F-A420-AD23418823F1

#### Campoletis

Förster, 1869

9C3E6BFE-2BBD-5C45-9734-6F170E2263AD

#### Campoletis agilis

(Holmgren, 1860)

A9FBD872-1B7A-549C-877B-878874055F80

##### Materials

- **Type status:** Other material. **Occurrence:** recordedBy: S. Cantone, A. Di Giulio; individualCount: 2; sex: 1 female, 1 male; occurrenceID: 5014D50B-EE81-5C07-A4AF-DC4A3380E0DD; **Location:** country: Italy; countryCode: IT; stateProvince: Sicily; municipality: Sortino, Siracusa; locality: Riserva Naturale di Pantalica; **Identification:** identifiedBy: F. Di Giovanni; dateIdentified: 2025; **Event:** eventDate: 05/05/2023; year: 2023; month: 5; day: 5; **Record Level:** institutionCode: FDGC
- **Type status:** Other material. **Occurrence:** recordedBy: S. Cantone, A. Di Giulio; individualCount: 5; sex: 1 female, 4 male; occurrenceID: 9A77AA4F-B4D3-5230-BADE-BD86E19E5544; **Location:** country: Italy; countryCode: IT; stateProvince: Sicily; municipality: Sortino, Siracusa; locality: Riserva Naturale di Pantalica; **Identification:** identifiedBy: F. Di Giovanni; dateIdentified: 2025; **Event:** eventDate: 17/05/2023; year: 2023; month: 5; day: 17; **Record Level:** institutionCode: FDGC
- **Type status:** Other material. **Occurrence:** recordedBy: S. Cantone, A. Di Giulio; individualCount: 1; sex: male; occurrenceID: 200E1545-1E6D-5B8C-B26D-A2CD1C907CD7; **Location:** country: Italy; countryCode: IT; stateProvince: Sicily; municipality: Sortino, Siracusa; locality: Riserva Naturale di Pantalica; **Identification:** identifiedBy: F. Di Giovanni; dateIdentified: 2025; **Event:** eventDate: 17/06/2023; year: 2023; month: 6; day: 17; **Record Level:** institutionCode: FDGC
- **Type status:** Other material. **Occurrence:** recordedBy: S. Cantone, A. Di Giulio; individualCount: 1; sex: male; occurrenceID: C704AC9C-4D4E-5846-80DC-B21786941996; **Location:** country: Italy; countryCode: IT; stateProvince: Sicily; municipality: Sortino, Siracusa; locality: Riserva Naturale di Pantalica; **Identification:** identifiedBy: F. Di Giovanni; dateIdentified: 2025; **Event:** eventDate: 09/07/2023; year: 2023; month: 7; day: 9; **Record Level:** institutionCode: FDGC

##### Distribution

Palaearctic (Eastern Palaearctic; Western Palaearctic) (Riedel 2017).

##### Notes

Species recorded for North and South Italy (Di Giovanni and Riedel 2017). This is the first record of the species for Sicily.

#### Campoletis annulata

(Gravenhorst, 1829)

B47409FA-EF66-5AA3-A8B0-7B42FAEB0F62

##### Materials

- **Type status:** Other material. **Occurrence:** recordedBy: S. Cantone, A. Di Giulio; individualCount: 1; sex: male; occurrenceID: 995FB2A6-D06B-5840-BA78-A525866D5A1C; **Location:** country: Italy; countryCode: IT; stateProvince: Sicily; municipality: Sortino, Siracusa; locality: Riserva Naturale di Pantalica; **Identification:** identifiedBy: F. Di Giovanni; dateIdentified: 2025; **Event:** eventDate: 27/04/2023; year: 2023; month: 4; day: 27; **Record Level:** institutionCode: FDGC

##### Distribution

Palaearctic (Eastern Palaearctic; Western Palaearctic) (Riedel 2017).

##### Notes

Species recorded for North and South Italy (Gribodo 1881, Zangheri 1969) and Sicily (Vidal 1997).

#### Campoletis latrator

(Gravenhorst, 1829)

E6032085-CB91-5C45-8142-FBA4A57337BB

##### Materials

- **Type status:** Other material. **Occurrence:** recordedBy: S. Cantone, A. Di Giulio; individualCount: 1; sex: female; occurrenceID: 9ED79705-3492-5687-8F17-D2682441D1F7; **Location:** country: Italy; countryCode: IT; stateProvince: Sicily; municipality: Sortino, Siracusa; locality: Riserva Naturale di Pantalica; **Identification:** identifiedBy: F. Di Giovanni; dateIdentified: 2025; **Event:** eventDate: 27/04/2023; year: 2023; month: 4; day: 27; **Record Level:** institutionCode: FDGC
- **Type status:** Other material. **Occurrence:** recordedBy: S. Cantone, A. Di Giulio; individualCount: 2; sex: females; occurrenceID: 778850DA-22B4-5926-B783-1888333957C2; **Location:** country: Italy; countryCode: IT; stateProvince: Sicily; municipality: Sortino, Siracusa; locality: Riserva Naturale di Pantalica; **Identification:** identifiedBy: F. Di Giovanni; dateIdentified: 2025; **Event:** eventDate: 05/05/2023; year: 2023; month: 5; day: 5; **Record Level:** institutionCode: FDGC
- **Type status:** Other material. **Occurrence:** recordedBy: S. Cantone, A. Di Giulio; individualCount: 7; sex: 4 females, 3 males; occurrenceID: 492B7101-032E-5DEB-94FB-767749C71CCE; **Location:** country: Italy; countryCode: IT; stateProvince: Sicily; municipality: Sortino, Siracusa; locality: Riserva Naturale di Pantalica; **Identification:** identifiedBy: F. Di Giovanni; dateIdentified: 2025; **Event:** eventDate: 17/05/2023; year: 2023; month: 5; day: 17; **Record Level:** institutionCode: FDGC
- **Type status:** Other material. **Occurrence:** recordedBy: S. Cantone, A. Di Giulio; individualCount: 1; sex: male; occurrenceID: D7A53BD2-40BB-5388-A691-258562004572; **Location:** country: Italy; countryCode: IT; stateProvince: Sicily; municipality: Sortino, Siracusa; locality: Riserva Naturale di Pantalica; **Identification:** identifiedBy: F. Di Giovanni; dateIdentified: 2025; **Event:** eventDate: 09/07/2023; year: 2023; month: 7; day: 9; **Record Level:** institutionCode: FDGC

##### Distribution

Palaearctic (Eastern Palaearctic; Western Palaearctic) (Riedel 2017).

##### Notes

Species recorded for North and South Italy (Gribodo 1881, Zangheri 1969) and Sicily (De Stefani 1895).

#### Cymodusa

Holmgren, 1859

C5640283-ECBA-50FD-8882-8F76B01E7DCB

#### Cymodusa (Cymodusa) australis

(Smits van Burgst, 1913)

571194BE-5AEC-5779-B990-FF36FB9790BC

##### Materials

- **Type status:** Other material. **Occurrence:** recordedBy: S. Cantone, A. Di Giulio; individualCount: 3; sex: 1 female, 2 males; occurrenceID: 4AB1A395-EB83-56E7-9001-312150804A3A; **Location:** country: Italy; countryCode: IT; stateProvince: Sicily; municipality: Sortino, Siracusa; locality: Riserva Naturale di Pantalica; **Identification:** identifiedBy: F. Di Giovanni; dateIdentified: 2025; **Event:** eventDate: 27/04/2023; year: 2023; month: 4; day: 27; **Record Level:** institutionCode: FDGC
- **Type status:** Other material. **Occurrence:** recordedBy: S. Cantone, A. Di Giulio; individualCount: 11; sex: 6 females, 5 males; occurrenceID: 05163467-23B7-52DF-B0E7-2D4BA5973F7E; **Location:** country: Italy; countryCode: IT; stateProvince: Sicily; municipality: Sortino, Siracusa; locality: Riserva Naturale di Pantalica; **Identification:** identifiedBy: F. Di Giovanni; dateIdentified: 2025; **Event:** eventDate: 05/05/2023; year: 2023; month: 5; day: 5; **Record Level:** institutionCode: FDGC
- **Type status:** Other material. **Occurrence:** recordedBy: S. Cantone, A. Di Giulio; individualCount: 3; sex: males; occurrenceID: F2F9FC4F-34D3-5B98-AE17-2C98B2F67EB0; **Location:** country: Italy; countryCode: IT; stateProvince: Sicily; municipality: Sortino, Siracusa; locality: Riserva Naturale di Pantalica; **Identification:** identifiedBy: F. Di Giovanni; dateIdentified: 2025; **Event:** eventDate: 17/05/2023; year: 2023; month: 5; day: 17; **Record Level:** institutionCode: FDGC
- **Type status:** Other material. **Occurrence:** recordedBy: S. Cantone, A. Di Giulio; individualCount: 2; sex: 1 female, 1 male; occurrenceID: 687E9EB1-7FBE-5A69-8371-C47582DE59F7; **Location:** country: Italy; countryCode: IT; stateProvince: Sicily; municipality: Sortino, Siracusa; locality: Riserva Naturale di Pantalica; **Identification:** identifiedBy: F. Di Giovanni; dateIdentified: 2025; **Event:** eventDate: 17/06/2023; year: 2023; month: 6; day: 17; **Record Level:** institutionCode: FDGC

##### Distribution

Palaearctic (Western Palaearctic).

##### Notes

Species recorded for North and South Italy (Scaramozzino 1995, Di Giovanni and Scaramozzino 2019). This is the first record of the species for Sicily.

#### Dusona

Cameron, 1901

6E9E7C80-625C-5409-BD82-D242D2C07A15

#### Dusona erythrogaster

(Förster, 1868)

5F02AF39-6A85-52FB-9567-952FD62D0843

##### Materials

- **Type status:** Other material. **Occurrence:** recordedBy: S. Cantone, A. Di Giulio; individualCount: 1; sex: female; occurrenceID: D1AFB1BA-4310-5D58-B123-4CB148866C05; **Location:** country: Italy; countryCode: IT; stateProvince: Sicily; municipality: Sortino, Siracusa; locality: Riserva Naturale di Pantalica; **Identification:** identifiedBy: F. Di Giovanni; dateIdentified: 2025; **Event:** eventDate: 05/05/2023; year: 2023; month: 5; day: 5; **Record Level:** institutionCode: FDGC

##### Distribution

Palaearctic (Western Palaearctic).

##### Notes

Species recorded for North and South Italy (Scaramozzino 1995, Pagliano 2009, Horstmann 2011). This is the first record of the species for Sicily.

#### Eriborus

Förster, 1869

5E0D802E-9CB4-5CEC-AE9C-F8A57B6B6873

#### Eriborus terebrator

Aubert, 1960

687EFD1A-D588-5F7A-AA77-1F6E840B2696

##### Materials

- **Type status:** Other material. **Occurrence:** recordedBy: S. Cantone, A. Di Giulio; individualCount: 1; sex: female; occurrenceID: CF942085-22A6-5215-BC49-8DFC2249ABFF; **Location:** country: Italy; countryCode: IT; stateProvince: Sicily; municipality: Sortino, Siracusa; locality: Riserva Naturale di Pantalica; **Identification:** identifiedBy: F. Di Giovanni; dateIdentified: 2025; **Event:** eventDate: 17/05/2023; year: 2023; month: 5; day: 17; **Record Level:** institutionCode: FDGC

##### Distribution

Palaearctic (Western Palaearctic).

##### Notes

Species recorded for North and South Italy (Di Giovanni and Reshchikov 2016, Di Giovanni and Scaramozzino 2019). This is the first record of the species for Sicily.

#### Nemeritis

Holmgren, 1860

AA6D2F9D-0BD3-5AAB-A055-98AC9C7A381C

#### Nemeritis fallax

(Gravenhorst, 1829)

BB0E618E-5380-5A0B-AF87-8734C8F3A08C

##### Materials

- **Type status:** Other material. **Occurrence:** recordedBy: S. Cantone, A. Di Giulio; individualCount: 1; sex: female; occurrenceID: 4A326684-AABE-5D68-815B-315CE8604364; **Location:** country: Italy; countryCode: IT; stateProvince: Sicily; municipality: Sortino, Siracusa; locality: Riserva Naturale di Pantalica; **Identification:** identifiedBy: F. Di Giovanni & Z. Vas; dateIdentified: 2025; **Event:** eventDate: 27/04/2023; year: 2023; month: 4; day: 27; **Record Level:** institutionCode: FDGC

##### Distribution

Palaearctic (Western Palaearctic).

##### Notes

**Figure 2.**
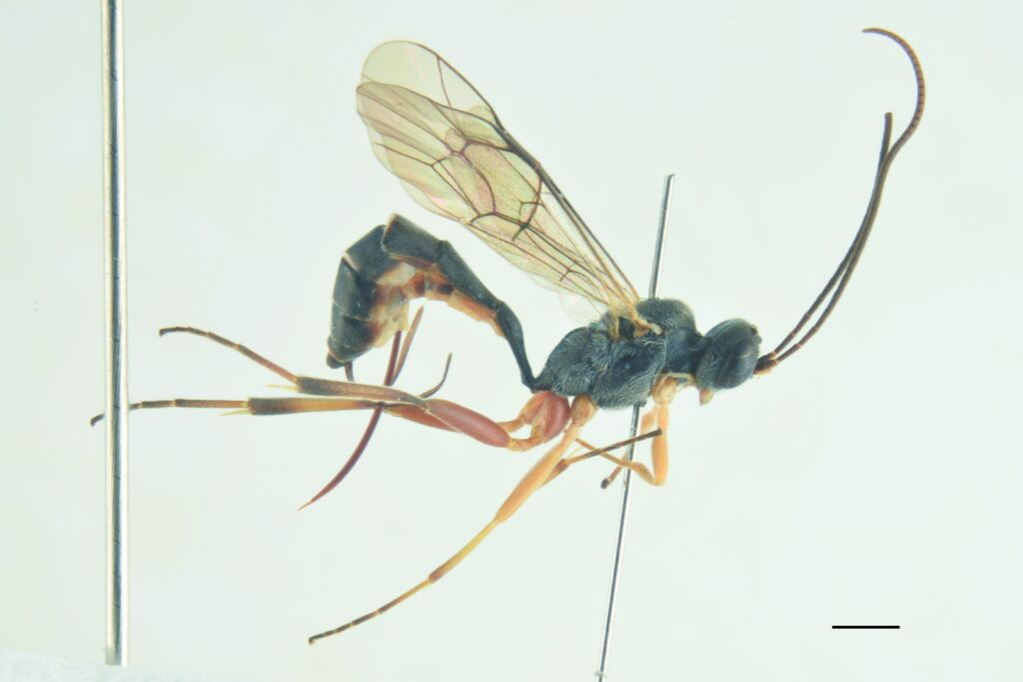
*Nemeritis fallax* (Gravenhorst, 1829), female, habitus. Scale bar 1 mm.

This is the first record of the species for Italy (Fig. 2).

#### Sinophorus

Förster, 1869

545C9DCB-D4DB-5F1B-8E89-509A49FE7E51

#### Sinophorus turionum

(Ratzeburg, 1844)

6D2CFC00-8FC9-5196-83F3-2BCAD6C953E3

##### Materials

- **Type status:** Other material. **Occurrence:** recordedBy: S. Cantone, A. Di Giulio; individualCount: 1; sex: female; occurrenceID: D40DAC5C-6E3E-5D87-ABFE-31D4401FCB86; **Location:** country: Italy; countryCode: IT; stateProvince: Sicily; municipality: Sortino, Siracusa; locality: Riserva Naturale di Pantalica; **Identification:** identifiedBy: F. Di Giovanni; dateIdentified: 2025; **Event:** eventDate: 17/05/2023; year: 2023; month: 5; day: 17; **Record Level:** institutionCode: FDGC

##### Distribution

Indomalyan; Nearctic (introduced); Palaearctic (Eastern Palaearctic; Western Palaearctic).

##### Notes

Species recorded in North and South Italy (Scaramozzino 1995). This is the first record of the species for Sicily.

#### Cryptinae

Kirby, 1837

EF275FC6-3675-5A6D-8EAF-A9C6A19D1FB2

#### Aptesini

Smith & Shenefelt, 1955

9ACA85B5-0401-55FF-8C8D-F442B63F671D

#### Aptesis

Förster, 1850

1F6E4055-C79E-56B1-98A6-2B76DDF4DB44

#### Aptesis nigrocincta

(Gravenhorst, 1815)

859A7631-C96F-5788-99D2-07BAFC58EC26

##### Materials

- **Type status:** Other material. **Occurrence:** recordedBy: S. Cantone, A. Di Giulio; individualCount: 1; sex: male; occurrenceID: 75148A22-40DD-5A68-A598-D65822C41BBE; **Location:** country: Italy; countryCode: IT; stateProvince: Sicily; municipality: Sortino, Siracusa; locality: Riserva Naturale di Pantalica; **Identification:** identifiedBy: F. Di Giovanni; dateIdentified: 2025; **Event:** eventDate: 27/04/2023; year: 2023; month: 4; day: 27; **Record Level:** institutionCode: FDGC
- **Type status:** Other material. **Occurrence:** recordedBy: S. Cantone, A. Di Giulio; individualCount: 1; sex: male; occurrenceID: E11E4184-E879-5A26-A074-A79F9931DDC0; **Location:** country: Italy; countryCode: IT; stateProvince: Sicily; municipality: Sortino, Siracusa; locality: Riserva Naturale di Pantalica; **Identification:** identifiedBy: F. Di Giovanni; dateIdentified: 2025; **Event:** eventDate: 05/05/2023; year: 2023; month: 5; day: 5; **Record Level:** institutionCode: FDGC
- **Type status:** Other material. **Occurrence:** recordedBy: S. Cantone, A. Di Giulio; individualCount: 1; sex: male; occurrenceID: 49A5BDE3-7F5E-5375-8BE2-8EE605D8FA64; **Location:** country: Italy; countryCode: IT; stateProvince: Sicily; municipality: Sortino, Siracusa; locality: Riserva Naturale di Pantalica; **Identification:** identifiedBy: F. Di Giovanni; dateIdentified: 2025; **Event:** eventDate: 17/05/2023; year: 2023; month: 5; day: 17; **Record Level:** institutionCode: FDGC

##### Distribution

Palaearctic (Eastern Palaearctic; Western Palaearctic).

##### Notes

Species recorded for North and South Italy (Gribodo 1881, Scaramozzino 1995, Pagliano 2009). This is the first record of the species for Sicily.

#### Cryptini

Kirby, 1837

68435D3A-6375-5EA6-98DE-F23EE93FD89C

#### Agrothereutes

Förster, 1850

725197CD-FF53-517C-B60A-74F6624519D1

#### Agrothereutes hospes

(Tschek, 1871)

42022221-7D3E-5C0C-AF4B-2BF1EB94B946

##### Materials

- **Type status:** Other material. **Occurrence:** recordedBy: S. Cantone, A. Di Giulio; individualCount: 2; sex: males; occurrenceID: 431730D6-EA8B-5D5A-96CB-8C0519D735DF; **Location:** country: Italy; countryCode: IT; stateProvince: Sicily; municipality: Sortino, Siracusa; locality: Riserva Naturale di Pantalica; **Identification:** identifiedBy: F. Di Giovanni; dateIdentified: 2025; **Event:** eventDate: 27/04/2023; year: 2023; month: 4; day: 27; **Record Level:** institutionCode: FDGC
- **Type status:** Other material. **Occurrence:** recordedBy: S. Cantone, A. Di Giulio; individualCount: 1; sex: male; occurrenceID: 73E88FBF-9866-5960-9B5A-D660A76A44DB; **Location:** country: Italy; countryCode: IT; stateProvince: Sicily; municipality: Sortino, Siracusa; locality: Riserva Naturale di Pantalica; **Identification:** identifiedBy: F. Di Giovanni; dateIdentified: 2025; **Event:** eventDate: 17/05/2023; year: 2023; month: 5; day: 17; **Record Level:** institutionCode: FDGC

##### Distribution

Palaearctic (Eastern Palaearctic; Western Palaearctic).

##### Notes

Species recorded for North and South Italy (Scaramozzino 1995). This is the first record of the species for Sicily.

#### Agrothereutes pumilus

(Kriechbaumer, 1899)

37A9109F-3E6C-54C6-8FE7-DB20B81397A7

##### Materials

- **Type status:** Other material. **Occurrence:** recordedBy: S. Cantone, A. Di Giulio; individualCount: 1; sex: male; occurrenceID: F546D58D-0AA7-51CB-9C48-F87344A99B0C; **Location:** country: Italy; countryCode: IT; stateProvince: Sicily; municipality: Sortino, Siracusa; locality: Riserva Naturale di Pantalica; **Identification:** identifiedBy: F. Di Giovanni; dateIdentified: 2025; **Event:** eventDate: 17/05/2023; year: 2023; month: 5; day: 17; **Record Level:** institutionCode: FDGC

##### Distribution

Palaearctic (Western Palaearctic).

##### Notes

Species recorded for North, South Italy and Sardinia (Gupta 1983, Delrio and Prota 1987, Scaramozzino 1995). This is the first record of the species for Sicily.

#### Enclisis

Townes, 1970

35B927D3-CA2A-5B3F-94E6-DB2879C199E1

#### Enclisis infernator

(Aubert, 1968)

DA24478E-A904-51A5-8BE3-EEFEA0637787

##### Materials

- **Type status:** Other material. **Occurrence:** recordedBy: S. Cantone, A. Di Giulio; individualCount: 2; sex: 1 female, 1 male; occurrenceID: C86C4B3B-6860-5C2F-B1D9-22BC4E02C87C; **Location:** country: Italy; countryCode: IT; stateProvince: Sicily; municipality: Sortino, Siracusa; locality: Riserva Naturale di Pantalica; **Identification:** identifiedBy: F. Di Giovanni; dateIdentified: 2025; **Event:** eventDate: 27/04/2023; year: 2023; month: 04; day: 27; **Record Level:** institutionCode: FDGC

##### Distribution

Palaearctic (Western Palaearctic).

##### Notes

Species recorded for North and South Italy (Schwarz 1989, Corcos et al. 2017). This is the first record of the species for Sicily.

#### Enclisis macilenta

(Gravenhorst, 1829)

13CDD7F8-3101-5183-A001-CEC4B1AE03DC

##### Materials

- **Type status:** Other material. **Occurrence:** recordedBy: S. Cantone, A. Di Giulio; individualCount: 1; sex: male; occurrenceID: D2F45EE9-8D68-5169-B2DA-F921AB2A1F59; **Location:** country: Italy; countryCode: IT; stateProvince: Sicily; municipality: Sortino, Siracusa; locality: Riserva Naturale di Pantalica; **Identification:** identifiedBy: F. Di Giovanni; dateIdentified: 2025; **Event:** eventDate: 05/05/2023; year: 2023; month: 5; day: 5; **Record Level:** institutionCode: FDGC

##### Distribution

Palaearctic (Western Palaearctic).

##### Notes

Species recorded for North Italy (Schwarz 1989) and Sicily (De Stefani 1895).

#### Enclisis vindex

(Tschek, 1871)

DA8FACD7-C476-52D8-86B4-25A3A855D979

##### Materials

- **Type status:** Other material. **Occurrence:** recordedBy: S. Cantone, A. Di Giulio; individualCount: 1; sex: female; occurrenceID: 074732A5-FC30-5550-96D4-3C19965353DB; **Location:** country: Italy; countryCode: IT; stateProvince: Sicily; municipality: Sortino, Siracusa; locality: Riserva Naturale di Pantalica; **Identification:** identifiedBy: F. Di Giovanni; dateIdentified: 2025; **Event:** eventDate: 09/07/2023; year: 2023; month: 7; day: 9; **Record Level:** institutionCode: FDGC

##### Distribution

Palaearctic (Western Palaearctic).

##### Notes

Species recorded for North and South Italy (Scaramozzino 1995, Corcos et al. 2017). This is the first record of the species for Sicily.

#### Hoplocryptus

Thomson, 1873

89B00B7A-B013-542A-A4C7-A8D70887F0A2

#### Hoplocryptus bellosus

(Curtis, 1837)

AEF78AC3-9478-5677-80A8-1266479988F3

##### Materials

- **Type status:** Other material. **Occurrence:** recordedBy: S. Cantone, A. Di Giulio; individualCount: 2; sex: females; occurrenceID: 5082ED79-7FB1-561A-9D95-455CF581A08F; **Location:** country: Italy; countryCode: IT; stateProvince: Sicily; municipality: Sortino, Siracusa; locality: Riserva Naturale di Pantalica; **Identification:** identifiedBy: F. Di Giovanni; dateIdentified: 2025; **Event:** eventDate: 09/07/2023; year: 2023; month: 7; day: 9; **Record Level:** institutionCode: FDGC
- **Type status:** Other material. **Occurrence:** recordedBy: S. Cantone, A. Di Giulio; individualCount: 1; sex: male; occurrenceID: A1B9EA87-8DFF-5BA1-A89F-87E7E4C580A7; **Location:** country: Italy; countryCode: IT; stateProvince: Sicily; municipality: Sortino, Siracusa; locality: Riserva Naturale di Pantalica; **Identification:** identifiedBy: F. Di Giovanni; dateIdentified: 2025; **Event:** eventDate: 12/08/2023; year: 2023; month: 8; day: 12; **Record Level:** institutionCode: FDGC

##### Distribution

Palaearctic (Eastern Palaearctic; Western Palaearctic).

##### Notes

Species present throughout Italy, including the major islands (Faggioli 1933, Grandi 1959, Scaramozzino 1995, Schwarz 2007).

#### Hoplocryptus odoriferator

(Dufour & Perris, 1840)

6BA9AF6E-DF5B-50E9-A863-18047733CFFF

##### Materials

- **Type status:** Other material. **Occurrence:** recordedBy: S. Cantone, A. Di Giulio; individualCount: 1; sex: female; occurrenceID: 6A29CF21-AE1C-58FA-AFE1-78A83905E3D9; **Location:** country: Italy; countryCode: IT; stateProvince: Sicily; municipality: Sortino, Siracusa; locality: Riserva Naturale di Pantalica; **Identification:** identifiedBy: F. Di Giovanni; dateIdentified: 2025; **Event:** eventDate: 05/05/2023; year: 2023; month: 5; day: 5; **Record Level:** institutionCode: FDGC
- **Type status:** Other material. **Occurrence:** recordedBy: S. Cantone, A. Di Giulio; individualCount: 2; sex: 1 female, 1 male; occurrenceID: C7E18497-4295-56BC-B5A1-070CA516E757; **Location:** country: Italy; countryCode: IT; stateProvince: Sicily; municipality: Sortino, Siracusa; locality: Riserva Naturale di Pantalica; **Identification:** identifiedBy: F. Di Giovanni; dateIdentified: 2025; **Event:** eventDate: 17/05/2023; year: 2023; month: 5; day: 17; **Record Level:** institutionCode: FDGC

##### Distribution

Palaearctic (Western Palaearctic).

##### Notes

Species present throughout Italy, including the major islands (Scaramozzino 1995, Schwarz 2007).

#### Myrmeleonostenus

Uchida, 1936

7858C6D7-28C8-568D-B8E8-EF6787388CF2

#### Myrmeleonostenus italicus

(Gravenhorst, 1829)

954BDA70-4A21-5B68-AE30-53EEEA6A1F7A

##### Materials

- **Type status:** Other material. **Occurrence:** recordedBy: S. Cantone, A. Di Giulio; individualCount: 1; sex: male; occurrenceID: DA6C88F0-A4E3-5925-BB80-3CB84CF4432E; **Location:** country: Italy; countryCode: IT; stateProvince: Sicily; municipality: Sortino, Siracusa; locality: Riserva Naturale di Pantalica; **Identification:** identifiedBy: F Di Giovanni; dateIdentified: 2025; **Event:** eventDate: 17/05/2023; year: 2023; month: 5; day: 17; **Record Level:** institutionCode: FDGC

##### Distribution

Palaearctic (Eastern Palaearctic; Western Palaearctic).

##### Notes

Species present throughout Italy, including the major islands (Scaramozzino 1995).

#### Schreineria

Schreiner, 1905

F737C997-B5D1-5775-B1A5-2729F87006EB

#### Schreineria populnea

(Giraud, 1872)

74FDB6A1-6AA9-55E7-86EF-1457301FB6D0

##### Materials

- **Type status:** Other material. **Occurrence:** recordedBy: S. Cantone, A. Di Giulio; individualCount: 1; sex: female; occurrenceID: 9ED45D78-091E-5B60-9710-F63F518986C3; **Location:** country: Italy; countryCode: IT; stateProvince: Sicily; municipality: Sortino, Siracusa; locality: Riserva Naturale di Pantalica; **Identification:** identifiedBy: F. Di Giovanni; dateIdentified: 2025; **Event:** eventDate: 17/05/2023; year: 2023; month: 5; day: 17; **Record Level:** institutionCode: FDGC
- **Type status:** Other material. **Occurrence:** recordedBy: S. Cantone, A. Di Giulio; individualCount: 1; sex: female; occurrenceID: A1AEAC94-AF55-589E-AB9B-4ED51B372132; **Location:** country: Italy; countryCode: IT; stateProvince: Sicily; municipality: Sortino, Siracusa; locality: Riserva Naturale di Pantalica; **Identification:** identifiedBy: F. Di Giovanni; dateIdentified: 2025; **Event:** eventDate: 09/07/2023; year: 2023; month: 7; day: 9; **Record Level:** institutionCode: FDGC
- **Type status:** Other material. **Occurrence:** recordedBy: S. Cantone, A. Di Giulio; individualCount: 1; sex: female; occurrenceID: 4E6449E9-6D5A-54E2-B318-0F3F77792AF8; **Location:** country: Italy; countryCode: IT; stateProvince: Sicily; municipality: Sortino, Siracusa; locality: Riserva Naturale di Pantalica; **Identification:** identifiedBy: F. Di Giovanni; dateIdentified: 2025; **Event:** eventDate: 12/08/2023; year: 2023; month: 8; day: 12; **Record Level:** institutionCode: FDGC

##### Distribution

Palaearctic (Eastern Palaearctic; Western Palaearctic).

##### Notes

Species recorded for North Italy (Scaramozzino 1995). This is the first record of the species for Sicily.

#### Stenarella

Szépligeti, 1916

726B9AE2-293C-5B74-97DE-C2600C074776

#### Stenarella domator

(Poda, 1761)

84EC5508-AD89-5D64-B249-75934CB60560

#### Stenarella domator domator

(Poda, 1761)

526F8D8D-4297-50FA-BAF7-3BBB48229F11

##### Materials

- **Type status:** Other material. **Occurrence:** recordedBy: S. Cantone, A. Di Giulio; individualCount: 1; sex: female; occurrenceID: 2721A004-3A0D-5CE7-A4F5-4B9FC93E5B93; **Location:** country: Italy; countryCode: IT; stateProvince: Sicily; municipality: Sortino, Siracusa; locality: Riserva Naturale di Pantalica; **Identification:** identifiedBy: F. Di Giovanni; dateIdentified: 2025; **Event:** eventDate: 05/05/2023; year: 2023; month: 5; day: 5; **Record Level:** institutionCode: FDGC

##### Distribution

Nearctic; Palaearctic (Eastern Palaearctic; Western Pelaearctic).

##### Notes

Recorded for North, South Italy and Sicily (De Stefani 1895, Scaramozzino 1995, Schwarz and Shaw 1998, Santos and Bordera 2025).

#### Trychosis

Förster, 1869

5EEB0945-BF7C-5796-8374-967A3DC0BCC3

#### Trychosis legator

(Thunberg, 1822)

D1D3BCA0-7722-5F6A-BD77-19CB29DD531D

##### Materials

- **Type status:** Other material. **Occurrence:** recordedBy: S. Cantone, A. Di Giulio; individualCount: 3; sex: females; occurrenceID: EA66DEE4-58B4-5FD8-8A45-00261F380A80; **Location:** country: Italy; countryCode: IT; stateProvince: Sicily; municipality: Sortino, Siracusa; locality: Riserva Naturale di Pantalica; **Identification:** identifiedBy: F. Di Giovanni; dateIdentified: 2025; **Event:** eventDate: 09/07/2023; year: 2023; month: 7; day: 9; **Record Level:** institutionCode: FDGC

##### Distribution

Palaearctic (Eastern Palaearctic; Western Palaearctic).

##### Notes

Species present throughout Italy, including the major islands (Scaramozzino 1995, Pagliano 2009).

#### Ctenopelmatinae

Förster, 1869

BE36DBA9-D913-59C1-AD05-6785D9A29017

#### Perilissini

Thomson, 1883

F76CFBD6-958E-5172-8442-F2DFC3E7DA79

#### Lathrolestes

Förster, 1869

D035F348-44BB-59A3-9E21-4F71FF406EF0

#### Lathrolestes luteolator

(Gravenhorst, 1829)

2049D94B-D703-5417-A287-7C2201FB1EC9

##### Materials

- **Type status:** Other material. **Occurrence:** recordedBy: S. Cantone, A. Di Giulio; individualCount: 1; sex: female; occurrenceID: 17743EFC-81E0-54DD-9146-BBC21221D396; **Location:** country: Italy; countryCode: IT; stateProvince: Sicily; municipality: Sortino, Siracusa; locality: Riserva Naturale di Pantalica; **Identification:** identifiedBy: F. Di Giovanni & A. Reshchikov; dateIdentified: 2025; **Event:** eventDate: 17/05/2023; year: 2023; month: 5; day: 17; **Record Level:** institutionCode: FDGC

##### Distribution

Australasian; Nearctic; Palaearctic (Western Palaearctic).

##### Notes

Species recorded for North Italy (Di Giovanni et al. 2015). This is the first record of the species for Sicily.

#### Perilissus

Holmgren, 1856

A8AE3E8E-74F8-51DF-9332-6B2DFC1EFBB6

#### Perilissus sericeus

(Gravenhorst, 1829)

4D7A084D-AF5D-556C-9B85-96CC505ED309

##### Materials

- **Type status:** Other material. **Occurrence:** recordedBy: S. Cantone, A. Di Giulio; individualCount: 1; sex: male; occurrenceID: E2817E8C-3D7F-5633-BA55-E818B1C91C4C; **Location:** country: Italy; countryCode: IT; stateProvince: Sicily; municipality: Sortino, Siracusa; locality: Riserva Naturale di Pantalica; **Identification:** identifiedBy: F. Di Giovanni & A. Reshchikov; dateIdentified: 2025; **Event:** eventDate: 27/04/2023; year: 2023; month: 4; day: 27; **Record Level:** institutionCode: FDGC

##### Distribution

Palaearctic (Western Palaearctic).

##### Notes

Species recorded for North Italy (Scaramozzino 1995, Pagliano 2009). This is the first record of the species for Sicily.

#### Diplazontinae

Viereck, 1918

2B22D9F6-BBA9-5843-8AF9-897242FE7B62

#### Diplazon

Nees, 1819

7A560749-9A09-5925-870B-69330C8BDADB

#### Diplazon laetatorius

(Fabricius, 1781)

85E32CC8-4953-5506-8364-C5684C07AC6B

##### Materials

- **Type status:** Other material. **Occurrence:** recordedBy: S. Cantone, A. Di Giulio; individualCount: 1; sex: female; occurrenceID: 3B393BE5-3383-5C0E-9746-E24D60AA7A5A; **Location:** country: Italy; countryCode: IT; stateProvince: Sicily; municipality: Sortino, Siracusa; locality: Riserva Naturale di Pantalica; **Identification:** identifiedBy: F. Di Giovanni; dateIdentified: 2025; **Event:** eventDate: 27/04/2023; year: 2023; month: 4; day: 27; **Record Level:** institutionCode: FDGC

##### Distribution

Worldwide.

##### Notes

Species present throughout Italy, including the major islands (Scaramozzino 1995).

#### Diplazon pectoratorius

(Thunberg, 1822)

A636C9CA-601D-5532-B3BD-C362E6B17765

##### Materials

- **Type status:** Other material. **Occurrence:** recordedBy: S. Cantone, A. Di Giulio; individualCount: 1; sex: male; occurrenceID: 2B2E2312-F764-5CED-94F4-999AF7EF821F; **Location:** country: Italy; countryCode: IT; stateProvince: Sicily; municipality: Sortino, Siracusa; locality: Riserva Naturale di Pantalica; **Identification:** identifiedBy: F. Di Giovanni; dateIdentified: 2025; **Event:** eventDate: 27/04/2023; year: 2023; month: 4; day: 27; **Record Level:** institutionCode: FDGC

##### Distribution

Palaearctic (Western Palaearctic).

##### Notes

Species recorded for North Italy (Scaramozzino 1995) and Sicily (Turrisi et al. 2007).

#### Syrphoctonus

Förster, 1869

FA89657C-D9D9-519A-BF17-1990B843DDBE

#### Syrphoctonus tarsatorius

(Panzer, 1809)

6F0B6DF1-BF99-53EF-8890-74F47B02C69F

##### Materials

- **Type status:** Other material. **Occurrence:** recordedBy: S. Cantone, A. Di Giulio; individualCount: 1; sex: male; occurrenceID: 6F3EF181-5A67-5F63-9CEE-985BF308E41E; **Location:** country: Italy; countryCode: IT; stateProvince: Sicily; municipality: Sortino, Siracusa; locality: Riserva Naturale di Pantalica; **Identification:** identifiedBy: F. Di Giovanni; dateIdentified: 2025; **Event:** eventDate: 17/05/2023; year: 2023; month: 5; day: 17; **Record Level:** institutionCode: FDGC

##### Distribution

Indomalayan; Nearctic; Palaearctic (Eastern Palaearctic; Western Palaearctic).

##### Notes

Species present throughout Italy, including the major islands (Scaramozzino 1995, Turrisi et al. 2007, Pagliano 2009).

#### Syrphophilus

Dasch, 1964

90D7DCF8-05CE-5426-BB43-588625366DB6

#### Syrphophilus bizonarius

(Gravenhorst, 1829)

3D89BCDE-784D-5FD7-AD71-43B04264101D

##### Materials

- **Type status:** Other material. **Occurrence:** recordedBy: S. Cantone, A. Di Giulio; individualCount: 1; sex: female; occurrenceID: 9BCC39D3-03C6-51EF-98A0-207CC3EACC92; **Location:** country: Italy; countryCode: IT; stateProvince: Sicily; municipality: Sortino, Siracusa; locality: Riserva Naturale di Pantalica; **Identification:** identifiedBy: F. Di Giovanni; dateIdentified: 2025; **Event:** eventDate: 05/05/2023; year: 2023; month: 05; day: 05; **Record Level:** institutionCode: FDGC

##### Distribution

Indomalayan; Nearctic; Palaearctic (Eastern Palaearctic; Western Palaearctic).

##### Horizon

Species present throughout Italy, including the major islands (Scaramozzino 1995, Turrisi et al. 2007, Pagliano 2009).

#### Ichneumoninae

Latreille, 1802

254B3CE4-C85D-5FBD-BCDB-B2DD95E3D83A

#### Ichneumonini

Latreille, 1802

C1B03F98-72DC-5812-A586-72162A60663E

#### Barichneumon

Thomson, 1893

48EFA266-4B84-5B75-BC72-53B48BFD4B01

#### Barichneumon derogator

(Wesmael, 1845)

B0DEF1C4-1E4E-50A6-81EC-940CF8430786

##### Materials

- **Type status:** Other material. **Occurrence:** recordedBy: S. Cantone, A. Di Giulio; individualCount: 1; sex: female; occurrenceID: 5C1A1471-74D6-5E88-99AA-77305D146CDF; **Location:** country: Italy; countryCode: IT; stateProvince: Sicily; municipality: Sortino, Siracusa; locality: Riserva Naturale di Pantalica; **Identification:** identifiedBy: F. Di Giovanni; dateIdentified: 2025; **Event:** eventDate: 17/05/2023; year: 2023; month: 5; day: 17; **Record Level:** institutionCode: FDGC

##### Distribution

Palaearctic (Eastern Palaearctic; Western Palaearctic).

##### Notes

Species recorded for North Italy (Scaramozzino 1995) and Sicily (De Stefani 1895, Riedel and Turrisi 2013).

#### Phaeogenini

Förster, 1869

925018FF-57CA-52B8-A17F-5EF0FE558688

#### Dilleritomus

Aubert, 1979

F78F8543-4DD7-5A64-A1A2-1A3F53C09EA2

#### Dilleritomus apertor

Aubert, 1979

794DDB27-B49F-577C-ABE0-5B3A87AC6F5E

##### Materials

- **Type status:** Other material. **Occurrence:** recordedBy: S. Cantone, A. Di Giulio; individualCount: 2; sex: males; occurrenceID: 5F7C4D17-A9DE-514A-8156-912E53A895B7; **Location:** country: Italy; countryCode: IT; stateProvince: Sicily; municipality: Sortino, Siracusa; locality: Riserva Naturale di Pantalica; **Identification:** identifiedBy: F. Di Giovanni; dateIdentified: 2025; **Event:** eventDate: 17/05/2023; year: 2023; month: 5; day: 17; **Record Level:** institutionID: FDGC

##### Distribution

Palaearctic (Western Palaearctic).

##### Notes

Species recorded for North Italy (Scaramozzino 1995). This is the first record of the species for Sicily.

#### Herpestomus

Wesmael, 1845

509A1165-F9D9-5A78-A638-29D2C92DA09F

#### Herpestomus arridens

(Gravenhorst, 1829)

AA9D57E7-A341-5A42-B7AC-EAC9709D1457

##### Materials

- **Type status:** Other material. **Occurrence:** recordedBy: S. Cantone, A. Di Giulio; individualCount: 1; sex: female; occurrenceID: 90D6F070-7D56-56DB-930F-0EE007E51733; **Location:** country: Italy; countryCode: IT; stateProvince: Sicily; municipality: Sortino, Siracusa; locality: Riserva Naturale di Pantalica; **Identification:** identifiedBy: F. Di Giovanni; dateIdentified: 2025; **Event:** eventDate: 17/05/2023; year: 2023; month: 5; day: 17; **Record Level:** institutionCode: FDGC

##### Distribution

Palaearctic (Eastern Palaearctic; Western Palaearctic).

##### Notes

Species recorded for North Italy (Scaramozzino 1995) and Sicily (Turrisi et al. 2007).

#### Misetus

Wesmael, 1845

0AA12DBB-B57C-557D-A261-3B92BB66D6FB

#### Misetus obscurus

Berthoumieu, 1897 stat. rev.

B8E6AFE4-9C56-51F3-91FE-4C66251B9B3C

##### Materials

- **Type status:** Other material. **Occurrence:** recordedBy: S. Cantone, A. Di Giulio; individualCount: 4; sex: males; occurrenceID: 68D20C7A-8527-53E4-A2C5-DBB7EAF25FE8; **Location:** country: Italy; countryCode: IT; stateProvince: Sicily; municipality: Sortino, Siracusa; locality: Riserva Naturale di Pantalica; **Identification:** identifiedBy: E. Diller; dateIdentified: 2024; **Event:** eventDate: 05/05/2023; year: 2023; month: 5; day: 5; **Record Level:** institutionCode: FDGC
- **Type status:** Other material. **Occurrence:** recordedBy: S. Cantone, A. Di Giulio; individualCount: 2; sex: 1 female, 1 male; occurrenceID: A0AAA00E-9BEA-5FAF-8B24-F8C09E14E57C; **Location:** country: Italy; countryCode: IT; stateProvince: Sicily; municipality: Sortino, Siracusa; locality: Riserva Naturale di Pantalica; **Identification:** identifiedBy: E. Diller; dateIdentified: 2024; **Event:** eventDate: 17/05/2023; year: 2023; month: 5; day: 17; **Record Level:** institutionCode: FDGC
- **Type status:** Other material. **Occurrence:** recordedBy: S. Cantone, A. Di Giulio; individualCount: 1; sex: female; occurrenceID: 9B3ADB04-2D4B-5AEF-95A9-E91B6203414C; **Location:** country: Italy; countryCode: IT; stateProvince: Sicily; municipality: Sortino, Siracusa; locality: Riserva Naturale di Pantalica; **Identification:** identifiedBy: E. Diller; dateIdentified: 2024; **Event:** eventDate: 09/07/2023; year: 2023; month: 7; day: 9; **Record Level:** institutionCode: FDGC

##### Distribution

Belgium, France (incl. Corsica), Romania, Sweden (but see Notes). This is the first record of the species for Italy.

##### Notes

According to Berthoumieu (1897), it is a colour form of *Misetus oculatus* Wesmael, 1845, characterszed by scape brown to black, thorax entirely black, hind coxae and sometimes also fore coxae black, hind legs and abdomen black, the latter with reddish margins on metasomal segments II–III. This form was later reported from Romania by Constantineanu (1965) and from Corsica by Aubert (1969); Constantineanu also cited it from France, Germany, Sweden and Lapland, repeating Berthoumieu's account, although the latter did not differentiate between the distribution of the two forms. Compared to the original description (Berthoumieu 1897), Aubert (1969) reports the scutellum as red and the hind legs as black, except for the yellow trochanters. In the specimens attributed to this form by Constantineanu (1965), the scutellum and postscutellum are black, whereas the hind femora are sometimes reddish-brown. According to Diller and Horstmann (1994), the *obscurus* form corresponds to *var. 1* reported by Wesmael (1845) for Belgium and in Holmgren (1890) for Sweden.

In the key to females of the Palaearctic species of the genus *Misetus* (Di Giovanni et al. 2018), the female of *M. obscurus* runs to the couplet 2, resembling *M. borealis* Kusigemati, 1974 and *M. nordicator* Selfa, 1995 in having a distinctly concave metasomal tergite V (Kusigemati 1974, Selfa and Diller 1995). However, it can be distinguished from both species by the number of flagellomeres (30), the occipital carina meeting the hypostomal carina at the base of the mandibles and the strigose-punctate postpetiole. Compared to the Mediterranean species (Figs 5, 6), *M. obscurus* exhibits a moderately pronounced median tooth on the clypeus (much more prominent in *M. oculatus*, barely visible in *M. strumiai* Di Giovanni, Scaramozzino & Diller, 2018), a distinctive microsculpture of postpetiole and metasomal tergite II, a yellowish scutellum (black in *M. hispanator* Selfa, 1995, red in *M. strumiai*) and a typically brownish-black hind coxa and femora (Selfa and Diller 1995, Di Giovanni et al. 2018).

Males are more difficult to distinguish and resemble *M. strumiai* males in colouration; they differ, however, by a more pronounced median tooth on the clypeus, antennae with a higher number of flagellomeres and tyloids (29-31 flagellomeres in *M. obscurus* vs. 24-25 in *M. strumiai*), less marked punctation on face, clypeus and metasomal tergite II and by the colouration of scutellum (black in males of *M. obscurus* and red in *M. strumiai*) (Di Giovanni et al. 2020) (Figs 7, 8).

Taxonomic identification was further supported by partial COX1 sequencing (GenBank accession PX272273). The closest matches in both the BOLD Systems v5 species identification and NCBI BLASTn search corresponded to *M. oculatus* (BOLD: GMFIE519-12; GenBank: MZ607881), with a p-distance of 5.41–5.84% (Table 1), indicating that the specimen cannot be confidently assigned to *M. oculatus*.

##### Diagnosis

**Figure 3.**
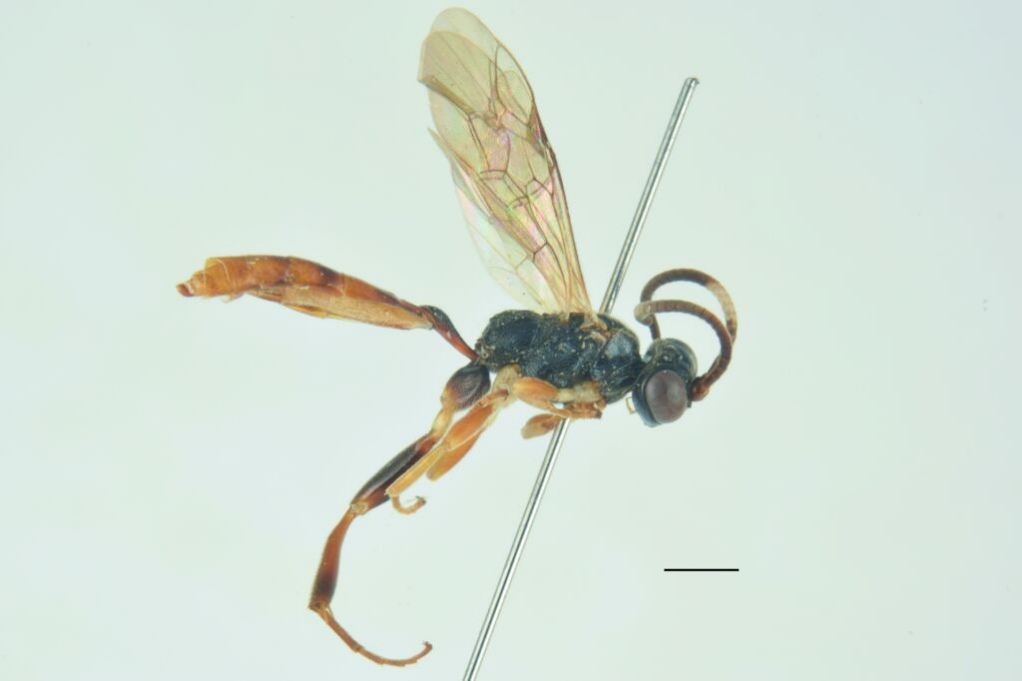
*Misetus obscurus* Berthoumieu, 1897, female, habitus. Scale bar 1 mm.

**Re-description, *Female*** (Fig. 3). Body length 7 mm. Fore wing length 4 mm.

Face about 0.5× as high as wide (width between compound eyes at the level of clypeal suture; height from antennal sockets to clypeal suture), polished with few scattered punctures centrally. Frons and vertex polished with very fine punctures. Clypeus polished and shining, with just few inconspicuous punctures, its apical margin almost straight and with a small developed medial tooth. Mandible slightly down-twisted at apex, with upper tooth longer than the lower one. Occipital carina complete, joining hypostomal carina at base of mandible. Antenna with 30 flagellomeres. Mesoscutum with dense punctures, notaulus deeply impressed in the anterior half; scutellum with few inconspicuous punctures, without lateral carinae. Mesopleuron, except for smooth speculum, covered with fine longitudinal striae, with a few distinct punctures interspersed in between; sternaulus impressed in the anterior half of mesopleuron; epicnemial carina present and reaching anterior margin of mesopleuron; posterior transverse carina of mesosternum interrupted at level of middle coxa. Fore wing areolet pentagonal, with cu-a opposite Rs&M. Hind wing with distal abscissa of Cu1 present. Coxa coriaceous and strongly punctate. Propodeum coriaceous, irregularly wrinkled, with propodeal carinae weakly marked, area superomedia slightly longer than wide; metapleuron smooth in the antero-dorsal half, with few scattered punctures, coriaceous and with irregular wrinkles on postero-ventral half. Metasoma covered with fine, short and sparse pubescence. Tergite I with dorsal longitudinal carinae weak, postpetiole strigose-punctate. Metasomal tergite II with thyridia weakly marked, slightly granulate basally, before thyridia, then slightly coriaceous medially and polished laterally and apically, with very few scattered punctures. Remaining metasomal tergites polished and without punctures; metasomal tergite V emarginate apically. Ovipositor short and upcurved.

**Colour**. Head black; antenna brownish-black with white markings on flagellomeres 5-9, scape and pedicel ventrally reddish; mandible, except for reddish teeth, yellowish-red; palps yellow. Mesosoma black; hind corner of pronotum, subtegular ridge, tegula and scutellum yellowish. Pterostigma yellowish-brown. Fore and mid-coxae and all trochanters yellow; fore and mid-femora and tibiae red; hind coxa black with yellow apex; trochanters yellow; hind femur black; hind tibia and tarsi reddish, tibia infuscate on apical third. Metasoma reddish-yellow; tergite I black, slightly reddish basally and apically; metasomal tergites II-III with a brownish median band. Ovipositor sheath red.

**Figure 4.**
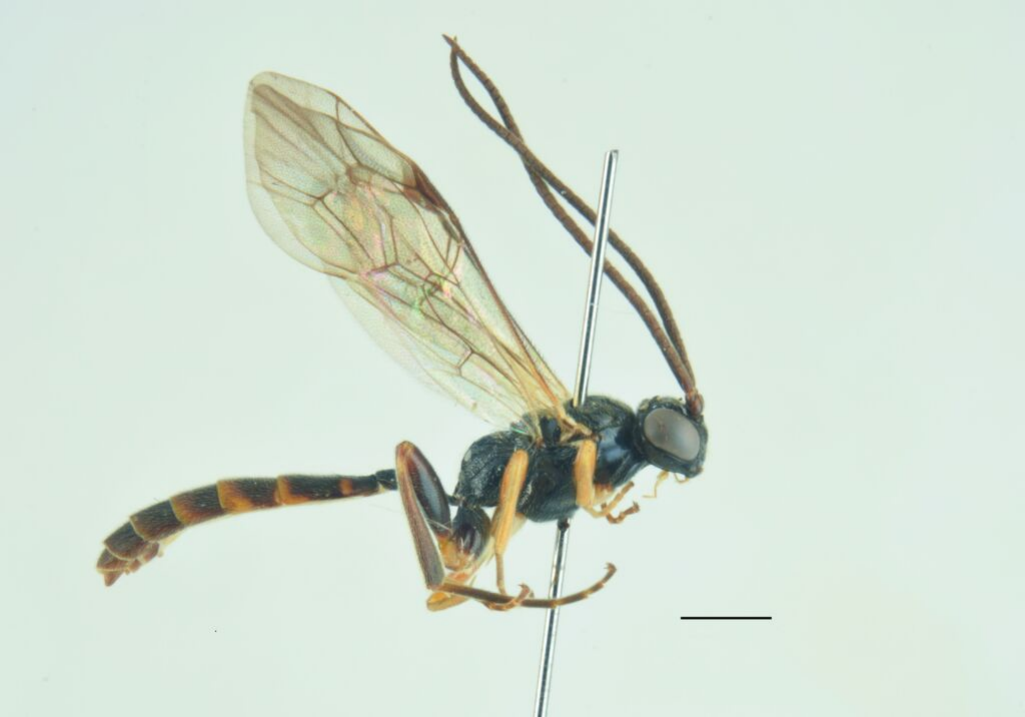
*Misetus obscurus* Berthoumieu, 1897, male, habitus. Scale bar 1 mm.

**Figure 5.**
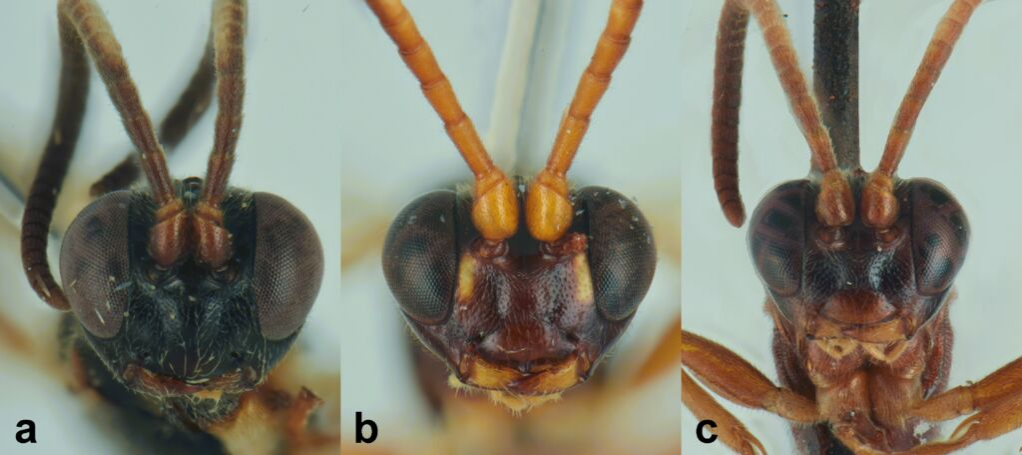
*Misetus* spp., females, face: **a)** *M. obscurus* Berthoumieu, 1897; **b)** *M. oculatus* Wesmael, 1845; **c)** *M. strumiai* Di Giovanni, Scaramozzino & Diller 2018.

**Figure 6.**
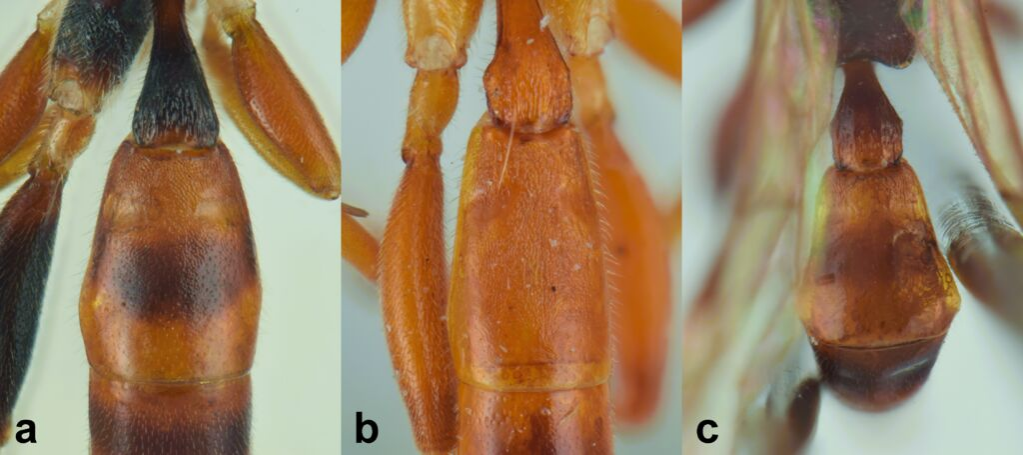
*Misetus* spp., females, metasomal tergites II: **a)** *M. obscurus* Berthoumieu, 1897; **b)** *M. oculatus* Wesmael, 1845; **c)** *M. strumiai* Di Giovanni, Scaramozzino & Diller 2018.

**Figure 7.**
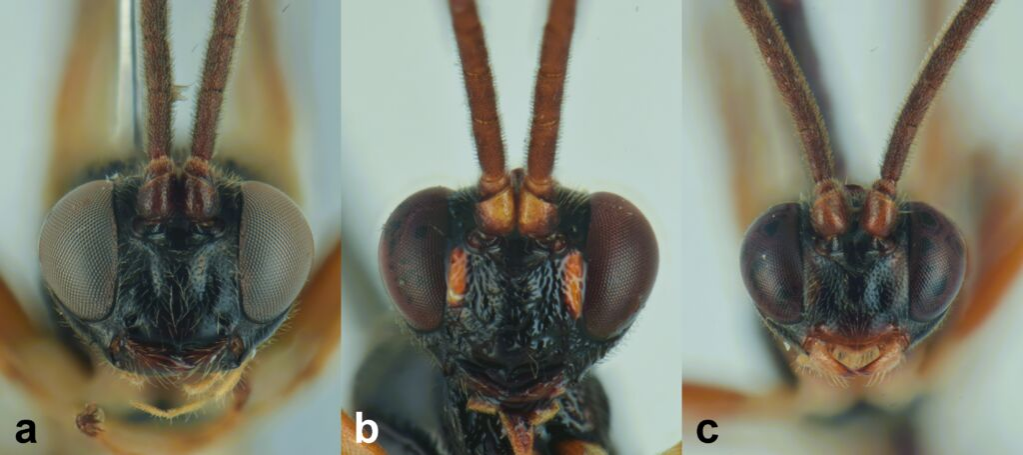
*Misetus* spp., males, face: **a)** *M. obscurus* Berthoumieu, 1897; **b)** *M. oculatus* Wesmael, 1845; **c)** *M. strumiai* Di Giovanni, Scaramozzino & Diller 2018.

**Figure 8.**
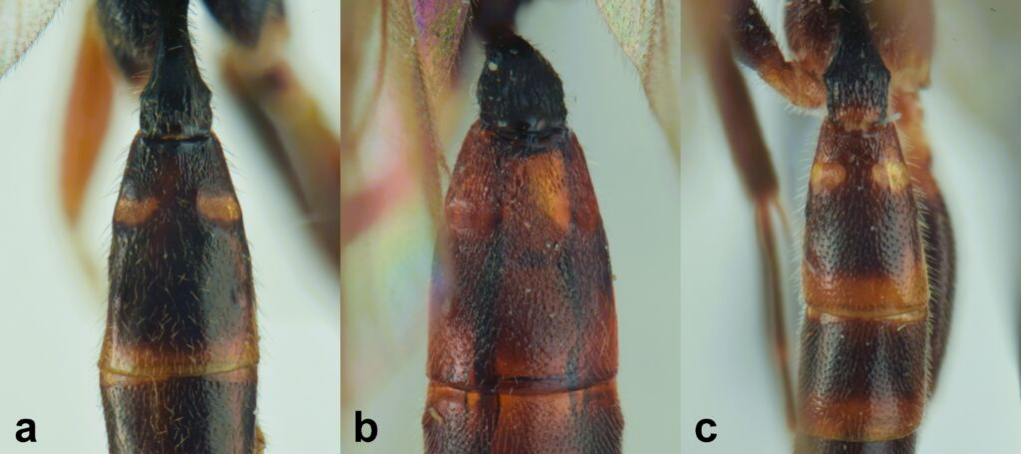
*Misetus* spp., males, metasomal tergite II: **a)** *M. obscurus* Berthoumieu, 1897; **b)** *M. oculatus* Wesmael, 1845; **c)** *M. strumiai* Di Giovanni, Scaramozzino & Diller 2018.

***Male*** (Fig. 4). As female, except for: antenna with 29-31 flagellomeres, tyloids on flagellar segments 7-15. Metasomal tergite II with thyridia more distinct, tergite roughly granulate basally, before thyridia, then slightly coriaceous medially and subpolished laterally and apically, with very few scattered punctures. In colour, similar to the female, except for mandible and scape brownish-black, scutellum black, hind trochanter black basally, yellow apically, hind tibia and tarsi darker, metasoma black with thyridia on metasomal tergite II and apical margin on tergites II-VI reddish.

#### Stenodontus

Berthoumieu, 1897

B4EE9EDB-230F-5E42-9D82-2C78D9E25C7F

#### Stenodontus meridionator

Aubert, 1959

A229B93D-077E-50AE-A6EB-FF9F7415AC8A

##### Materials

- **Type status:** Other material. **Occurrence:** recordedBy: S. Cantone, A. Di Giulio; individualCount: 1; sex: female; occurrenceID: C75B63BD-77FB-5267-A0A8-29CB5F166D21; **Location:** country: Italy; countryCode: IT; stateProvince: Sicily; municipality: Sortino, Siracusa; locality: Riserva Naturale di Pantalica; **Identification:** identifiedBy: F. Di Giovanni; dateIdentified: 2025; **Event:** eventDate: 17/05/2023; year: 2023; month: 5; day: 17; **Record Level:** institutionCode: FDGC
- **Type status:** Other material. **Occurrence:** recordedBy: S. Cantone, A. Di Giulio; individualCount: 1; sex: male; occurrenceID: EE6AD95C-0912-5DBA-86E7-321B91C521C4; **Location:** country: Italy; countryCode: IT; stateProvince: Sicily; municipality: Sortino, Siracusa; locality: Riserva Naturale di Pantalica; **Identification:** identifiedBy: F. Di Giovanni; dateIdentified: 2025; **Event:** eventDate: 09/07/2023; year: 2023; month: 7; day: 9; **Record Level:** institutionCode: FDGC
- **Type status:** Other material. **Occurrence:** recordedBy: S. Cantone, A. Di Giulio; individualCount: 4; sex: 1 female, 3 males; occurrenceID: 315E05A8-62AF-5D43-A63E-E3BF3D00CF59; **Location:** country: Italy; countryCode: IT; stateProvince: Sicily; municipality: Sortino, Siracusa; locality: Riserva Naturale di Pantalica; **Identification:** identifiedBy: F. Di Giovanni; dateIdentified: 2025; **Event:** eventDate: 12/08/2023; year: 2023; month: 8; day: 12; **Record Level:** institutionCode: FDGC

##### Distribution

Palaearctic (Western Palaearctic).

##### Notes

Species recorded for South Italy and Sardinia (Diller 1993, Scaramozzino 1995). This is the first record of the species for Sicily.

#### Platylabini

Berthoumieu, 1904

787BAF51-E07E-5328-B61D-FF8D172274B3

#### Cyclolabus

Heinrich, 1936

FFC48777-A3E4-5204-8F93-A65E513A3B57

#### Cyclolabus axillatorius

(Thunberg, 1822)

F582F094-926D-52B0-B95C-49E87B1C744B

##### Materials

- **Type status:** Other material. **Occurrence:** recordedBy: S. Cantone, A. Di Giulio; individualCount: 4; sex: males; occurrenceID: 2E62E411-242C-5131-93F2-2F9BCC15FD39; **Location:** country: Italy; countryCode: IT; stateProvince: Sicily; municipality: Sortino, Siracusa; locality: Riserva Naturale di Pantalica; **Identification:** identifiedBy: F. Di Giovanni; dateIdentified: 2025; **Event:** eventDate: 27/04/2023; year: 2023; month: 4; day: 27; **Record Level:** institutionCode: FGDC
- **Type status:** Other material. **Occurrence:** recordedBy: S. Cantone, A. Di Giulio; individualCount: 4; sex: females; occurrenceID: FB3F98EE-A6C9-59CF-B16A-414CAC9B3720; **Location:** country: Italy; countryCode: IT; stateProvince: Sicily; municipality: Sortino, Siracusa; locality: Riserva Naturale di Pantalica; **Identification:** identifiedBy: F. Di Giovanni; dateIdentified: 2025; **Event:** eventDate: 05/05/2023; year: 2023; month: 5; day: 5; **Record Level:** institutionCode: FGDC

##### Distribution

Palaearctic (Eastern Palaearctic; Western Palaearctic).

##### Notes

Species present throughout Italy, including the major islands (Scaramozzino 1995).

#### Platylabus

Wesmael, 1845

A511FAD5-13A1-5982-A773-BAF6B6BD7455

#### Platylabus rufator

Riedel, 2012

D3B977FA-6AD4-5285-A28E-5B5B3F7498DE

##### Materials

- **Type status:** Other material. **Occurrence:** recordedBy: S. Cantone, A. Di Giulio; individualCount: 2; sex: females; occurrenceID: CD7066CE-DA59-5ECE-854A-FC384E1511C3; **Location:** country: Italy; countryCode: IT; stateProvince: Sicily; municipality: Sortino, Siracusa; locality: Riserva Naturale di Pantalica; **Identification:** identifiedBy: F. Di Giovanni; dateIdentified: 2025; **Event:** eventDate: 27/04/2023; year: 2023; month: 4; day: 27; **Record Level:** institutionCode: FDGC
- **Type status:** Other material. **Occurrence:** recordedBy: S. Cantone, A. Di Giulio; individualCount: 5; sex: 4 females, 1 male; occurrenceID: E0647D37-5269-50ED-9084-636FDF299AF4; **Location:** country: Italy; countryCode: IT; stateProvince: Sicily; municipality: Sortino, Siracusa; locality: Riserva Naturale di Pantalica; **Identification:** identifiedBy: F. Di Giovanni; dateIdentified: 2025; **Event:** eventDate: 05/05/2023; year: 2023; month: 5; day: 5; **Record Level:** institutionCode: FDGC

##### Distribution

The species is only known from Sicily (Riedel and Turrisi 2013).

##### Notes

The species has been described on a single female from Mt. Etna (Riedel and Tomarchio 2012, Riedel and Turrisi 2013). *Platylabus rufator* was described by Riedel and Tomarchio (2012); however, the work has since been retracted. According to the ICZN (1999: Article 8.8), the retraction does not affect the availability of the new name published in the original work. Therefore, the authorship of *P. rufator* remains Riedel, 2012.

##### Diagnosis

**Figure 9.**
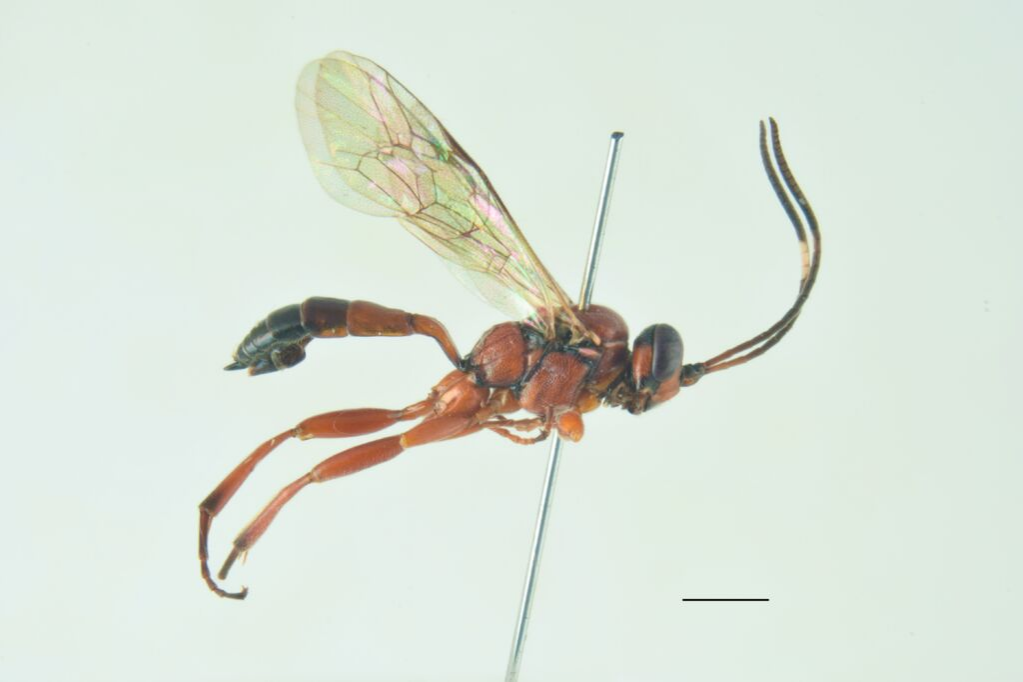
*Platylabus rufator* Riedel, 2012, female, habitus. Scale bar 1 mm.

***Female*** (Fig. 9). With respect to the original description (Riedel and Tomarchio 2012), antenna with 27-28 flagellomeres, white markings on flagellomeres (7)8-11(12). Face black with only a reddish-yellow spot in the middle or more extensive reddish-yellow colouration, also covering the clypeus and sometimes gena. Usually metasomal tergites I-III red, but sometimes metasomal tergite III brown or metasomal tergite IV almost enterely red. Hind tibia sometimes blackish in the apical 0.3-0.4.

**Figure 10.**
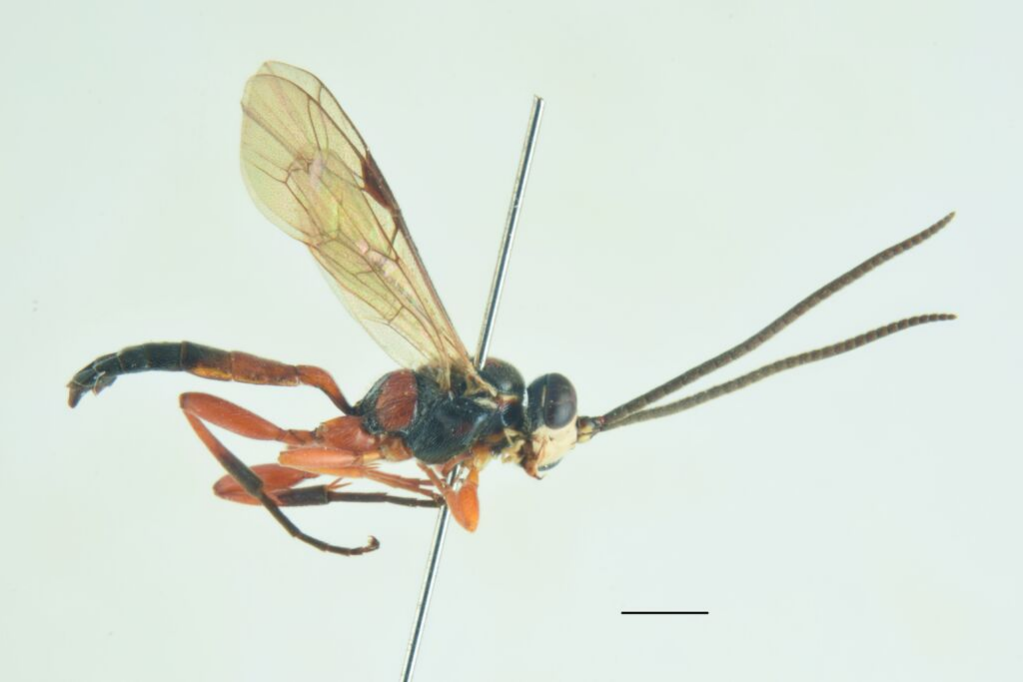
*Platylabus rufator* Riedel, 2012, male, habitus. Scale bar 1 mm.

**Figure 11.**
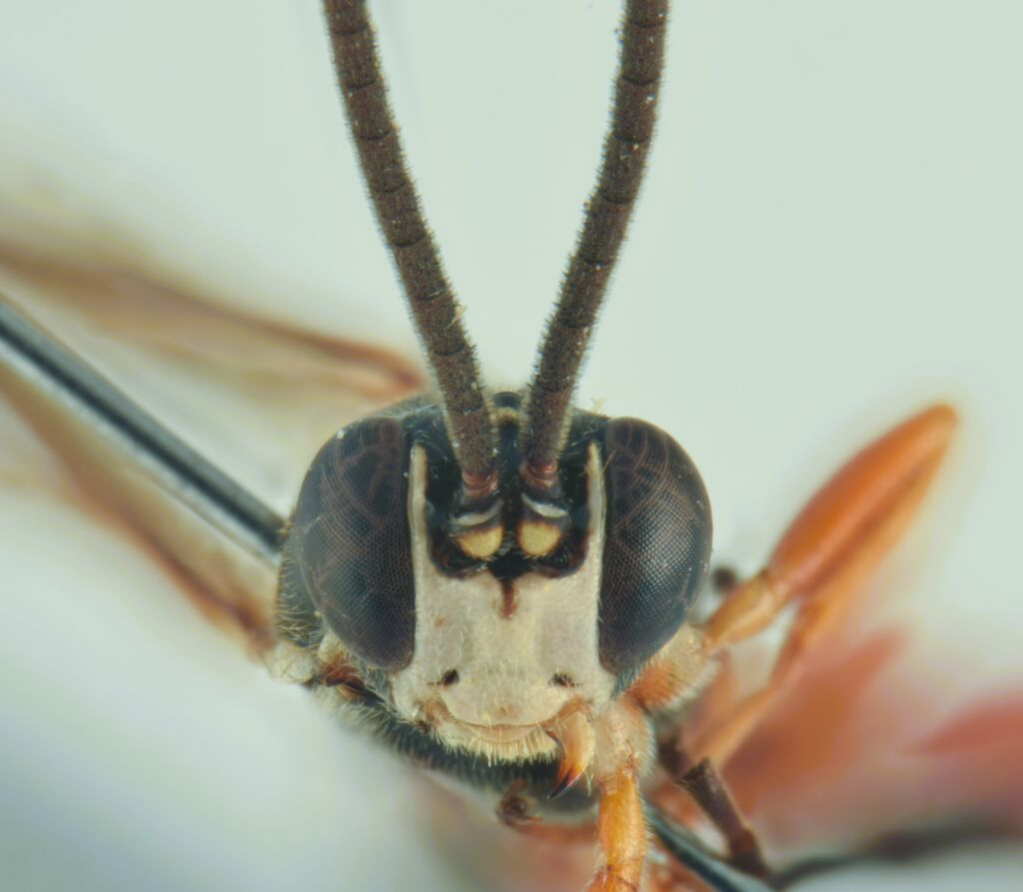
*Platylabus rufator* Riedel, 2012, male, face, frontal view.

***Description of the male*** (Figs 10, 11): Similar to female, but with the following differences: antenna stouter than female, linear, with 29 segments, without tyloids; face, including clypeus, mandibles (except for reddish-brown teeth), inner orbits and spots on outer orbits ivory; scape yellow ventrally; antenna brown, without white markings; mesosoma and metasoma more extensively black, with faint reddish colouration; apical area of propodeum black; hind tibia black in the apical 0.5.

#### Mesochorinae

Förster, 1869

831B2EE5-F2FB-5BDB-9640-F3AE0BC5852A

#### Astiphromma

Förster, 1869

F6ADF07C-AF83-5F3E-95BC-1E1469557C05

#### Astiphromma italicum

Schwenke, 1999

F02558AE-F447-5941-A640-2A4D9AEBCF73

##### Materials

- **Type status:** Other material. **Occurrence:** recordedBy: S. Cantone, A. Di Giulio; individualCount: 1; sex: female; occurrenceID: DD41D31E-68E0-5C41-ACAB-1A2B512982F1; **Location:** country: Italy; countryCode: IT; stateProvince: Sicily; municipality: Sortino, Siracusa; locality: Riserva Naturale di Pantalica; **Identification:** identifiedBy: F. Di Giovanni; dateIdentified: 2025; **Event:** eventDate: 17/05/2023; year: 2023; month: 5; day: 17; **Record Level:** institutionCode: FDGC

##### Distribution

Palaearctic (Western Palaearctic).

##### Notes

Species recorded for North and South Italy (Schwenke 1999). This is the first record of the species for Sicily.

#### Astiphromma splenium

(Curtis, 1833)

E0EF9A95-DE17-5169-BCA2-A4AD05317E06

##### Materials

- **Type status:** Other material. **Occurrence:** recordedBy: S. Cantone, A. Di Giulio; individualCount: 2; sex: males; occurrenceID: 57169ADE-532C-580B-9070-594AABF4DA2E; **Location:** country: Italy; countryCode: IT; stateProvince: Sicily; municipality: Sortino, Siracusa; locality: Riserva Naturale di Pantalica; **Identification:** identifiedBy: F. Di Giovanni & M. Riedel; dateIdentified: 2025; **Event:** eventDate: 27/04/2023; year: 2023; month: 4; day: 27; **Record Level:** institutionCode: FDGC
- **Type status:** Other material. **Occurrence:** recordedBy: S. Cantone, A. Di Giulio; individualCount: 1; sex: female; occurrenceID: 73336059-2E30-553F-87F0-FCC6C807BCC1; **Location:** country: Italy; countryCode: IT; stateProvince: Sicily; municipality: Sortino, Siracusa; locality: Riserva Naturale di Pantalica; **Identification:** identifiedBy: F. Di Giovanni; dateIdentified: 2025; **Event:** samplingProtocol: Malaise trap; eventDate: 05/05/2023; year: 2023; month: 5; day: 5; **Record Level:** institutionCode: FDGC
- **Type status:** Other material. **Occurrence:** recordedBy: S. Cantone, A. Di Giulio; individualCount: 1; sex: male; occurrenceID: C83E1823-E325-594D-92CA-77020D96D939; **Location:** country: Italy; countryCode: IT; stateProvince: Sicily; municipality: Sortino, Siracusa; locality: Riserva Naturale di Pantalica; **Identification:** identifiedBy: F. Di Giovanni & M. Riedel; dateIdentified: 2025; **Event:** eventDate: 17/05/2023; year: 2023; month: 5; day: 17; **Record Level:** institutionCode: FDGC
- **Type status:** Other material. **Occurrence:** recordedBy: S. Cantone, A. Di Giulio; individualCount: 1; sex: male; occurrenceID: 3C8E5A9C-762D-5150-B869-93D377D218E0; **Location:** country: Italy; countryCode: IT; stateProvince: Sicily; municipality: Sortino, Siracusa; locality: Riserva Naturale di Pantalica; **Identification:** identifiedBy: F. Di Giovanni & M. Riedel; dateIdentified: 2025; **Event:** eventDate: 09/07/2023; year: 2023; month: 7; day: 9; **Record Level:** institutionCode: FDGC

##### Distribution

Nearctic; Palaearctic (Eastern Palaearctic, Western Palaearctic).

##### Notes

Species recorded for North Italy (Scaramozzino 1995). This is the first record of the species for Sicily.

#### Metopiinae

Förster, 1869

7A0D33A7-AD51-5762-A0D5-0B9A55CBC72E

#### Exochus

Gravenhorst, 1829

46B03A02-1703-5EC7-8652-47A74EFEC157

#### Exochus semilividus

van Vollenhoven, 1875

38653891-9B59-565C-8957-491E404D5C78

##### Materials

- **Type status:** Other material. **Occurrence:** recordedBy: S. Cantone, A. Di Giulio; individualCount: 1; sex: female; occurrenceID: 47F32E61-560D-5BB2-AF6A-E92961E131E5; **Location:** country: Italy; countryCode: IT; stateProvince: Sicily; municipality: Sortino, Siracusa; locality: Riserva Naturale di Pantalica; **Identification:** identifiedBy: F. Di Giovanni & N. Johansson; dateIdentified: 2025; **Event:** eventDate: 27/04/2023; year: 2023; month: 4; day: 27; **Record Level:** institutionCode: FDGC
- **Type status:** Other material. **Occurrence:** recordedBy: S. Cantone, A. Di Giulio; individualCount: 2; sex: males; occurrenceID: A0BE0B03-87BD-51DC-BD82-CBC9864CCBE0; **Location:** country: Italy; countryCode: IT; stateProvince: Sicily; municipality: Sortino, Siracusa; locality: Riserva Naturale di Pantalica; **Identification:** identifiedBy: F. Di Giovanni & N. Johansson; dateIdentified: 2025; **Event:** eventDate: 05/05/2023; year: 2023; month: 5; day: 5; **Record Level:** institutionCode: FDGC
- **Type status:** Other material. **Occurrence:** recordedBy: S. Cantone, A. Di Giulio; individualCount: 1; sex: male; occurrenceID: ABA04E05-3A23-557D-A416-280259B6D61D; **Location:** country: Italy; countryCode: IT; stateProvince: Sicily; municipality: Sortino, Siracusa; locality: Riserva Naturale di Pantalica; **Identification:** identifiedBy: F. Di Giovanni & N. Johansson; dateIdentified: 2025; **Event:** eventDate: 17/06/2023; year: 2023; month: 6; day: 17; **Record Level:** institutionCode: FDGC

##### Distribution

Palaearctic (Eastern Palaearctic; Western Palaearctic) (Johansson and Astafurova 2025).

##### Notes

Species recorded for North Italy (Scaramozzino 1995). This is the first record of the species for Sicily.

#### Exochus thomsoni

Schmiedeknecht, 1924

BC832CD7-6415-5574-B54B-91248CC3EED7

##### Materials

- **Type status:** Other material. **Occurrence:** recordedBy: S. Cantone, A. Di Giulio; individualCount: 1; sex: male; occurrenceID: 5952FC50-C6AE-5ECC-A0B7-F59153500BBF; **Location:** country: Italy; countryCode: IT; stateProvince: Sicily; municipality: Sortino, Siracusa; locality: Riserva Naturale di Pantalica; **Identification:** identifiedBy: F. Di Giovanni; dateIdentified: 2025; **Event:** eventDate: 05/05/2023; year: 2023; month: 5; day: 5; **Record Level:** institutionCode: FDGC
- **Type status:** Other material. **Occurrence:** recordedBy: S. Cantone, A. Di Giulio; individualCount: 3; sex: 2 females, 1 male; occurrenceID: FA48B331-2C25-5A23-A35B-A254B1D6949C; **Location:** country: Italy; countryCode: IT; stateProvince: Sicily; municipality: Sortino, Siracusa; locality: Riserva Naturale di Pantalica; **Identification:** identifiedBy: F. Di Giovanni; dateIdentified: 2025; **Event:** eventDate: 17/05/2023; year: 2023; month: 5; day: 17; **Record Level:** institutionCode: FDGC
- **Type status:** Other material. **Occurrence:** recordedBy: S. Cantone, A. Di Giulio; individualCount: 1; sex: female; occurrenceID: 83C79768-02C4-5CD8-927C-4147F71BABA6; **Location:** country: Italy; countryCode: IT; stateProvince: Sicily; municipality: Sortino, Siracusa; locality: Riserva Naturale di Pantalica; **Identification:** identifiedBy: F. Di Giovanni; dateIdentified: 2025; **Event:** eventDate: 17/05/2023; year: 2023; month: 6; day: 17; **Record Level:** institutionCode: FDGC
- **Type status:** Other material. **Occurrence:** recordedBy: S. Cantone, A. Di Giulio; individualCount: 2; sex: males; occurrenceID: 44A145DF-0222-5006-97D0-D652958B1250; **Location:** country: Italy; countryCode: IT; stateProvince: Sicily; municipality: Sortino, Siracusa; locality: Riserva Naturale di Pantalica; **Identification:** identifiedBy: F. Di Giovanni; dateIdentified: 2025; **Event:** eventDate: 17/05/2023; year: 2023; month: 8; day: 21; **Record Level:** institutionCode: FDGC

##### Distribution

Palaearctic (Eastern Pelaearctic; Western Palaearctic).

##### Notes

Species reported for Italy, without regional details (Johansson and Astafurova 2025) and for Sicily as *Exochus humerator* Aubert, 1960 (Scaramozzino 1995).

#### Hypsicera

Latreille, 1829

780F3D12-78F3-5FE7-8599-94A13F7681DE

#### Hypsicera britannica

Tolkanitz, 2011

0E0AC642-BF06-5EA7-97BA-7D9D6281505E

##### Materials

- **Type status:** Other material. **Occurrence:** recordedBy: S. Cantone, A. Di Giulio; individualCount: 1; sex: female; occurrenceID: 1560F494-ED94-52BA-BB29-CA86765D529F; **Location:** country: Italy; countryCode: IT; stateProvince: Sicily; municipality: Sortino, Siracusa; locality: Riserva Naturale di Pantalica; **Identification:** identifiedBy: F. Di Giovanni; dateIdentified: 2025; **Event:** eventDate: 05/05/2023; year: 2023; month: 5; day: 5; **Record Level:** institutionCode: FDGC

##### Distribution

Palaearctic (Western Palaearctic).

##### Notes

Species recorded for North Italy (Scaramozzino 1995). This is the first record of the species for Sicily.

#### Ophioninae

Shuckard, 1840

2E4165A4-CB04-5C0F-BE6F-7D2396810AED

#### Enicospilus

Stephens, 1835

E425BB2D-95F3-5D52-9955-F0B886A5AA07

#### Enicospilus inflexus

(Ratzeburg, 1844)

68B2AEB2-4534-5C73-816E-76617ADB7022

##### Materials

- **Type status:** Other material. **Occurrence:** recordedBy: S. Cantone, A. Di Giulio; individualCount: 1; sex: female; occurrenceID: 87305CE9-E143-50EB-94DE-04C7575CB9D2; **Location:** country: Italy; countryCode: IT; stateProvince: Sicily; municipality: Sortino, Siracusa; locality: Riserva Naturale di Pantalica; **Identification:** identifiedBy: F. Di Giovanni; dateIdentified: 2025; **Event:** eventDate: 05/05/2023; year: 2023; month: 5; day: 5; **Record Level:** institutionCode: FDGC

##### Distribution

Palaearctic (Eastern Palaearctic; Western Palaearctic).

##### Notes

Species recorded for North, South Italy and Sicily (Scaramozzino 1995).

#### Enicospilus marocator

Aubert, 1982

5EBBFA5D-31D8-5C36-942B-6CCAED079456

##### Materials

- **Type status:** Other material. **Occurrence:** recordedBy: S. Cantone, A. Di Giulio; individualCount: 1; sex: female; occurrenceID: 01AF9D25-CB92-52B1-B7A3-AC11FD4676ED; **Location:** country: Italy; countryCode: IT; stateProvince: Sicily; municipality: Sortino, Siracusa; locality: Riserva Naturale di Pantalica; **Identification:** identifiedBy: F. Di Giovanni & N. Johansson; dateIdentified: 2025; **Event:** eventDate: 21/08/2023; year: 2023; month: 8; day: 21; **Record Level:** institutionCode: FDGC

##### Distribution

Palaearctic (Western Palaearctic).

##### Notes

**Figure 12.**
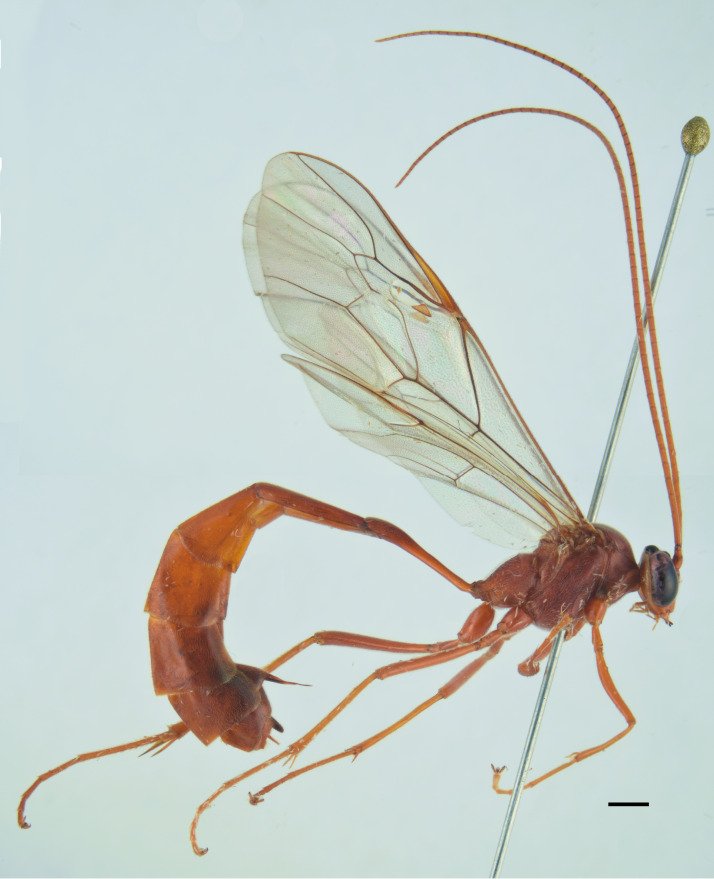
*Enicospilus marocator* Aubert, 1986, female, habitus. Scale bar 1 mm.

Species known from Morocco and Spain (Aubert 1982, Izquierdo 1982, Izquierdo 1984). This is the first record of the species for Italy (Fig. 12).

#### Ophion

Fabricius, 1798

88E2B64B-F12E-57FD-A7AF-28F9F5056B7B

#### Ophion castilloae

Johansson, 2021

06A9C64C-ADED-5951-B1ED-0E4A0CDE98D1

##### Materials

- **Type status:** Other material. **Occurrence:** recordedBy: S. Cantone, A. Di Giulio; individualCount: 1; sex: female; occurrenceID: 1D2F8FFE-869C-5744-B248-24A4E9B36E4E; **Location:** country: Italy; countryCode: IT; stateProvince: Sicily; municipality: Sortino, Siracusa; locality: Riserva Naturale di Pantalica; **Identification:** identifiedBy: F. Di Giovanni & N. Johansson; dateIdentified: 2025; **Event:** eventDate: 05/05/2023; year: 2023; month: 5; day: 5; **Record Level:** institutionCode: FDGC

##### Distribution

Palaearctic (Western Palaearctic) (Johansson 2021).

##### Notes

**Figure 13.**
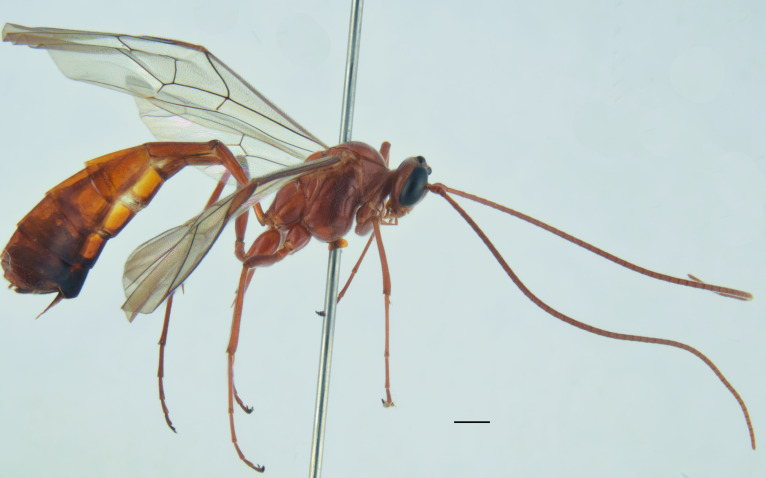
*Ophion castilloae* Johansson, 2021, female, habitus. Scale bar 1 mm.

Species described from Andorra, Croatia, Greece, Portugal and Spain (Johansson 2021). This is the first record of the species for Italy (Fig. 13).

#### Ophion longigena

Thomson, 1888

DC58D040-A901-5120-97DA-1011CDFFC8CB

##### Materials

- **Type status:** Other material. **Occurrence:** recordedBy: S. Cantone, A. Di Giulio; individualCount: 1; sex: female; occurrenceID: 90874DBC-2231-55EE-87D8-09626A94B689; **Location:** country: Italy; countryCode: IT; stateProvince: Sicily; municipality: Sortino, Siracusa; locality: Riserva Naturale di Pantalica; **Identification:** identifiedBy: F. Di Giovanni; dateIdentified: 2025; **Event:** eventDate: 05/05/2023; year: 2023; month: 5; day: 5; **Record Level:** institutionCode: FDGC

##### Distribution

Palaearctic (Eastern Palaearctic; Western Palaearctic).

##### Notes

Species recorded for North Italy and Sardinia (Scaramozzino 1995). This is the first record of the species for Sicily.

#### Ophion mediterraneus

Johansson, 2021

28F50969-5A1F-528C-A947-4FC4A09B8CA2

##### Materials

- **Type status:** Other material. **Occurrence:** recordedBy: S. Cantone, A. Di Giulio; individualCount: 1; sex: male; occurrenceID: 8C7B1570-EED4-5108-B3EC-C59B257E2AB0; **Location:** country: Italy; countryCode: IT; stateProvince: Sicily; municipality: Sortino, Siracusa; locality: Riserva Naturale di Pantalica; **Identification:** identifiedBy: F. Di Giovanni & N. Johansson; dateIdentified: 2025; **Event:** eventDate: 05/05/2023; year: 2023; month: 5; day: 5; **Record Level:** institutionCode: FDGC

##### Distribution

Palaearctic (Western Palaearctic) (Johansson 2021).

##### Notes

**Figure 14.**
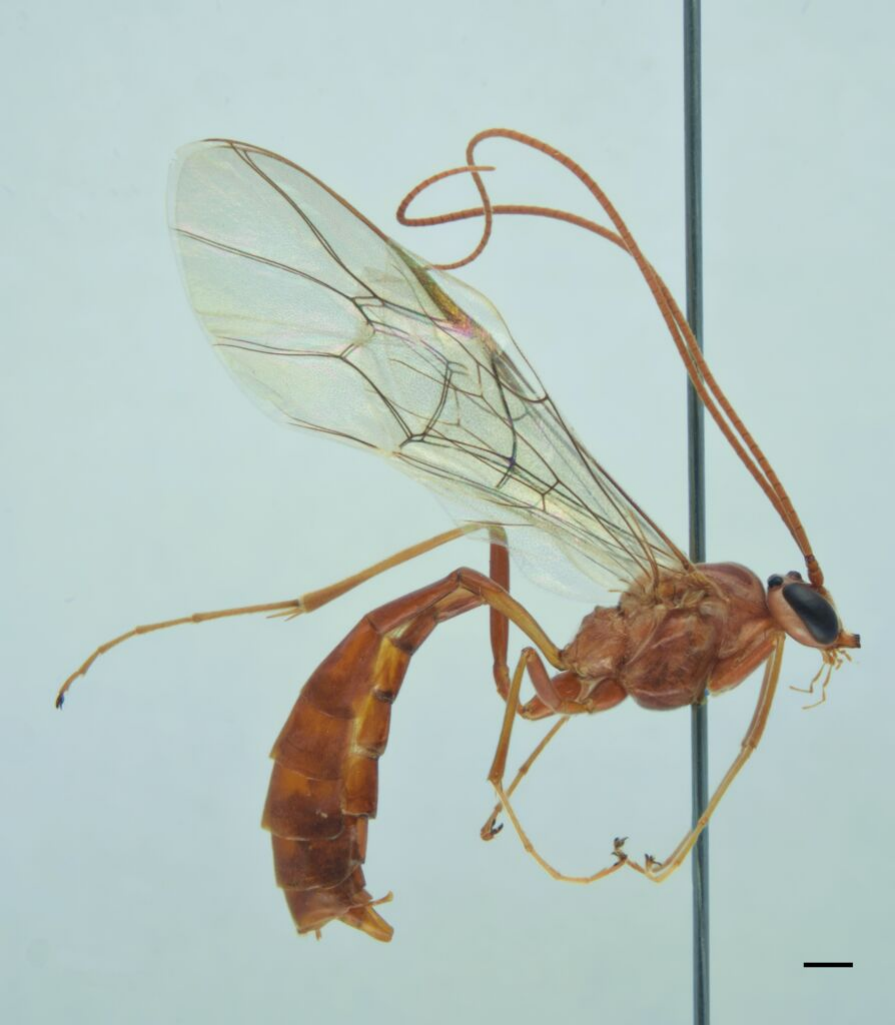
*Ophion mediterraneus* Johansson, 2021, male, habitus. Scale bar 1 mm.

Species described from Andorra, Morocco and Spain (Johansson 2021). This is the first record of the species for Italy (Fig. 14).

#### Ophion mocsaryi

Brauns, 1889

39838DB0-1EF8-5CCB-808B-C0F2FF16C57C

##### Materials

- **Type status:** Other material. **Occurrence:** recordedBy: S. Cantone, A. Di Giulio; individualCount: 1; sex: female; occurrenceID: 4151044D-59C4-55CA-83FB-63E35E5AAAC9; **Location:** country: Italy; countryCode: IT; stateProvince: Sicily; municipality: Sortino, Siracusa; locality: Riserva Naturale di Pantalica; **Identification:** identifiedBy: F. Di Giovanni; dateIdentified: 2025; **Event:** eventDate: 05/05/2023; year: 2023; month: 5; day: 5; **Record Level:** institutionCode: FDGC

##### Distribution

Palaearctic (Eastern Palaearctic; Western Palaearctic).

##### Notes

Species recorded for North, South Italy and Sardinia (Scaramozzino 1995). This is the first record of the species for Sicily.

#### Orthocentrinae

Förster, 1869

A65C29AC-BA8C-5DD7-A2FE-C66A00F1DB07

#### Helictes

Haliday, 1837

F2CE7EE0-6FAD-5C6E-B647-CA0A32756C86

#### Helictes borealis

(Holmgren, 1857)

24930D15-EBE2-5AEB-8328-3B97E2793915

##### Materials

- **Type status:** Other material. **Occurrence:** recordedBy: S. Cantone, A. Di Giulio; individualCount: 1; sex: male; occurrenceID: EC36F746-A938-58E3-8D9E-BA9DA4561FAA; **Location:** country: Italy; countryCode: IT; stateProvince: Sicily; municipality: Sortino, Siracusa; locality: Riserva Naturale di Pantalica; **Identification:** identifiedBy: F. Di Giovanni; dateIdentified: 2025; **Event:** eventDate: 05/05/2023; year: 2023; month: 5; day: 5; **Record Level:** institutionCode: FDGC

##### Distribution

Nearctic; Palaearctic (Eastern Palaearctic; Western Palaearctic).

##### Notes

Species recorded for North Italy (Scaramozzino 1995). This is the first record of the species for Sicily.

#### Helictes meridionator

Aubert, 1961

30CF7C54-39F3-5DBD-A5B1-81896C75C828

##### Materials

- **Type status:** Other material. **Occurrence:** recordedBy: S. Cantone, A. Di Giulio; individualCount: 1; sex: male; occurrenceID: B899AF4C-368E-5B18-ABCC-C6FA428B44CD; **Location:** country: Italy; countryCode: IT; stateProvince: Sicily; municipality: Sortino, Siracusa; locality: Riserva Naturale di Pantalica; **Identification:** identifiedBy: O. Varga; dateIdentified: 2025; **Event:** eventDate: 27/04/2023; year: 2023; month: 4; day: 27; **Record Level:** institutionCode: FDGC
- **Type status:** Other material. **Occurrence:** recordedBy: S. Cantone, A. Di Giulio; individualCount: 1; sex: male; occurrenceID: 0936ACCD-63D1-582D-8E15-A09699256CB3; **Location:** country: Italy; countryCode: IT; stateProvince: Sicily; municipality: Sortino, Siracusa; locality: Riserva Naturale di Pantalica; **Identification:** identifiedBy: F. Di Giovanni; dateIdentified: 2025; **Event:** eventDate: 05/05/2023; year: 2023; month: 5; day: 5; **Record Level:** institutionCode: FDGC
- **Type status:** Other material. **Occurrence:** recordedBy: S. Cantone, A. Di Giulio; individualCount: 1; sex: male; occurrenceID: CB2B8DD9-7317-545D-B97F-D6C96E81DC82; **Location:** country: Italy; countryCode: IT; stateProvince: Sicily; municipality: Sortino, Siracusa; locality: Riserva Naturale di Pantalica; **Identification:** identifiedBy: O. Varga; dateIdentified: 2025; **Event:** eventDate: 09/07/2023; year: 2023; month: 7; day: 9; **Record Level:** institutionCode: FDGC

##### Distribution

Palaearctic (Eastern Palaearctic; Western Palaearctic).

##### Notes

**Figure 15.**
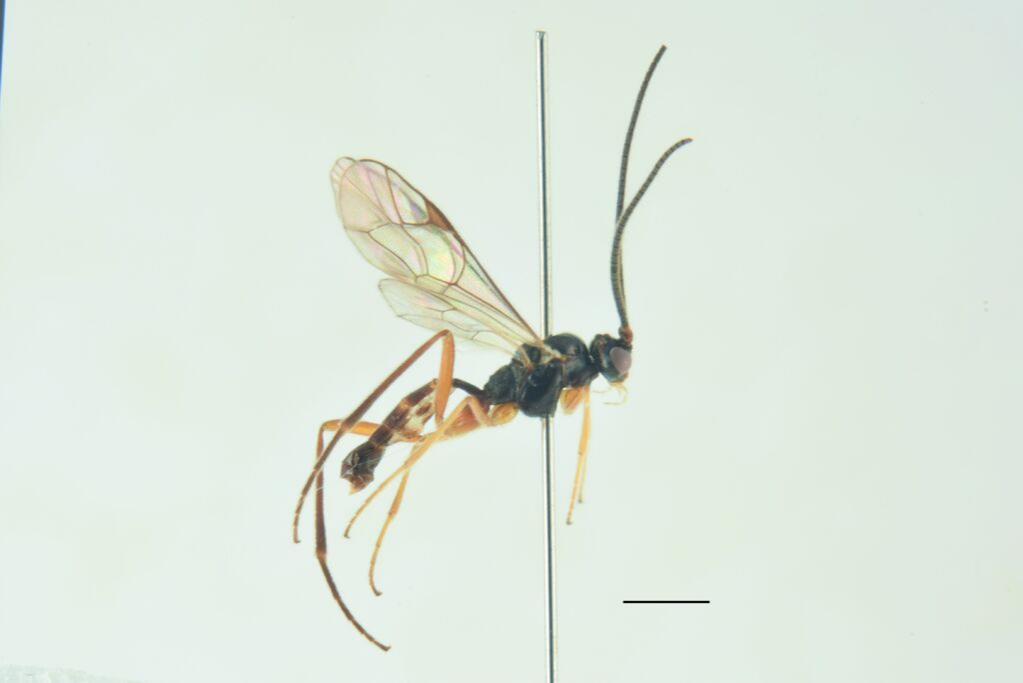
*Helictes meridionator* Aubert, 1961, male, habitus. Scale bar 1 mm.

Species recorded for France (incl. Corsica), Kazakhstan and Russia (Aubert 1961, Aubert 1963, Humala 2007). This is the first record of the species for Italy (Fig. 15).

#### Orthocentrus

Gravenhorst, 182

BDC03DBA-8C1E-5675-A6DB-D5AFB319E4E2

#### Orthocentrus asper

(Gravenhorst, 1829)

BE2E8A84-7162-5051-9DA5-3169EDD2E3CB

##### Materials

- **Type status:** Other material. **Occurrence:** recordedBy: S. Cantone, A. Di Giulio; individualCount: 1; sex: female; occurrenceID: 8E5F6D11-BAE7-58AE-B3BB-B841B0689197; **Location:** country: Italy; countryCode: IT; stateProvince: Sicily; municipality: Sortino, Siracusa; locality: Riserva Naturale di Pantalica; **Identification:** identifiedBy: O. Varga; dateIdentified: 2025; **Event:** eventDate: 27/04/2023; year: 2023; month: 4; day: 27; **Record Level:** institutionCode: FDGC
- **Type status:** Other material. **Occurrence:** recordedBy: S. Cantone, A. Di Giulio; individualCount: 1; sex: female; occurrenceID: FFE80D11-25D7-5DB1-B5E4-F30277B23851; **Location:** country: Italy; countryCode: IT; stateProvince: Sicily; municipality: Sortino, Siracusa; locality: Riserva Naturale di Pantalica; **Identification:** identifiedBy: O. Varga; dateIdentified: 2025; **Event:** eventDate: 17/05/2023; year: 2023; month: 5; day: 1; **Record Level:** institutionCode: FDGC

##### Distribution

Nearctic; Palaearctic (Eastern Palaearctic; Western Palaearctic).

##### Notes

Species recorded for North Italy (Scaramozzino 1995). This is the first record of the species for Sicily.

#### Orthocentrus fulvipes

Gravenhorst, 1829

1DF469C4-6FF1-5157-8777-20614A0D7869

##### Materials

- **Type status:** Other material. **Occurrence:** recordedBy: S. Cantone, A. Di Giulio; individualCount: 1; sex: male; occurrenceID: 709DB177-2EC4-504B-B63C-D2B1EC248240; **Location:** country: Italy; countryCode: IT; stateProvince: Sicily; municipality: Sortino, Siracusa; locality: Riserva Naturale di Pantalica; **Identification:** identifiedBy: O. Varga; dateIdentified: 2025; **Event:** eventDate: 17/06/2023; year: 2023; month: 6; day: 17; **Record Level:** institutionCode: FDGC

##### Distribution

Indomalayan; Palaearctic (Eastern Palaearctic; Western Palaearctic).

##### Notes

Species recorded for North and South Italy (Scaramozzino 1995; misidentified as *O. strigatus* Holmgren, 1858 in Corcos et al. (2017) and Di Giovanni and Scaramozzino (2019)). This is the first record of the species for Sicily.

#### Orthocentrus protervus

Holmgren, 1858

59797845-514A-5738-88DB-8F7AC9C024F4

##### Materials

- **Type status:** Other material. **Occurrence:** recordedBy: S. Cantone, A. Di Giulio; individualCount: 2; sex: 1 female, 1 male; occurrenceID: 07EED37A-F953-5F16-919C-3857114A3020; **Location:** country: Italy; countryCode: IT; stateProvince: Sicily; municipality: Sortino, Siracusa; locality: Riserva Naturale di Pantalica; **Identification:** identifiedBy: O. Varga; dateIdentified: 2025; **Event:** eventDate: 05/05/2023; year: 2023; month: 5; day: 5; **Record Level:** institutionCode: FDGC

##### Distribution

Palaearctic (Eastern Palaearctic; Western Palaearctic).

##### Notes

**Figure 16.**
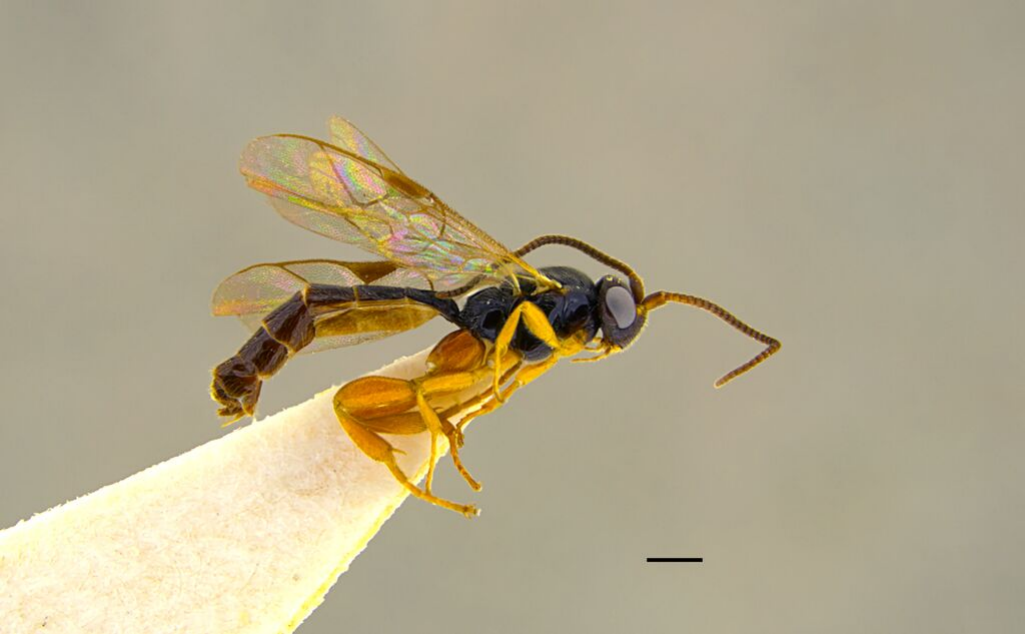
*Orthocentrus protervus* Holmgren, 1858, female, habitus. Scale bar 0.5 mm.

**Figure 17.**
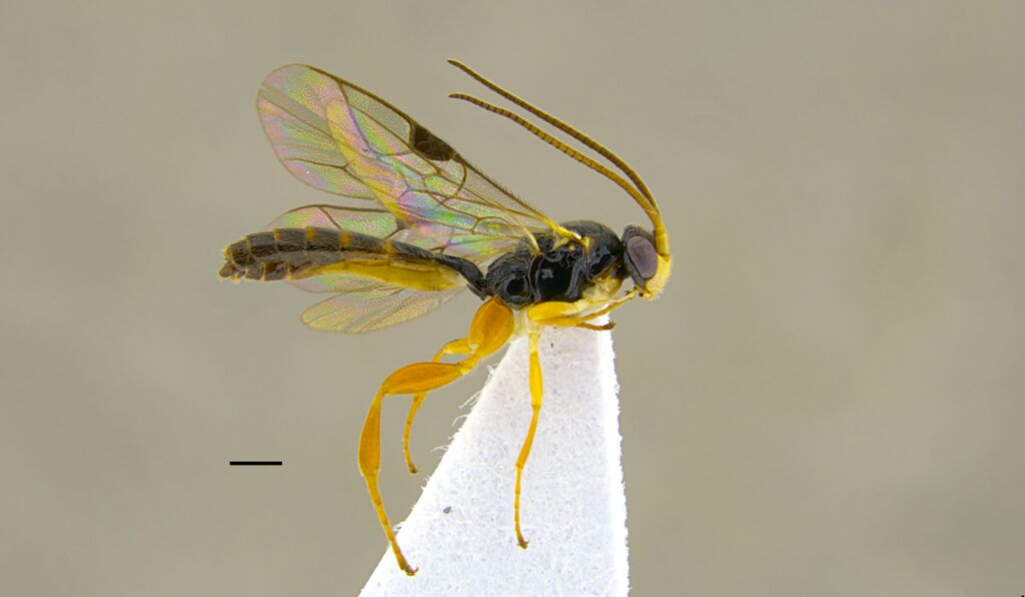
*Orthocentrus protervus* Holmgren, 1858, male, habitus. Scale bar 0.5 mm.

This is the first record of the species for Italy (Figs 16, 17).

#### Oxytorinae

Thomson, 1883

00A6F3B9-53B7-544C-B24F-DEF54827C837

#### Oxytorus

Förster, 1869

8A2E1C98-9D63-5E30-8979-F9A2D9115E8C

#### Oxytorus armatus

Thomson, 1883

AE4D17DF-94B7-57F8-9162-5979F3F002B5

##### Materials

- **Type status:** Other material. **Occurrence:** recordedBy: S. Cantone, A. Di Giulio; individualCount: 2; sex: males; occurrenceID: 26FAACD8-3A66-5817-B911-888228B31BC9; **Location:** country: Italy; countryCode: IT; stateProvince: Sicily; municipality: Sortino, Siracusa; locality: Riserva Naturale di Pantalica; **Identification:** identifiedBy: F. Di Giovanni; dateIdentified: 2025; **Event:** eventDate: 17/05/2023; year: 2023; month: 5; day: 17; **Record Level:** institutionCode: FDGC
- **Type status:** Other material. **Occurrence:** recordedBy: S. Cantone, A. Di Giulio; individualCount: 1; sex: males; occurrenceID: 912668FA-A7B9-5B25-94DB-1EC7B023ABA7; **Location:** country: Italy; countryCode: IT; stateProvince: Sicily; municipality: Sortino, Siracusa; locality: Riserva Naturale di Pantalica; **Identification:** identifiedBy: F. Di Giovanni; dateIdentified: 2025; **Event:** eventDate: 17/06/2023; year: 2023; month: 6; day: 17; **Record Level:** institutionCode: FDGC

##### Distribution

Palaearctic (Western Palaearctic).

##### Notes

Species recorded from North and South Italy (Scaramozzino 1995, Corcos et al. 2017). This is the first record of the subfamily Oxytorinae and the species for Sicily.

#### Oxytorus luridator

(Gravenhorst, 1820)

EA368055-433F-52F3-A3CB-3C22E7B70651

##### Materials

- **Type status:** Other material. **Occurrence:** recordedBy: S. Cantone, A. Di Giulio; individualCount: 4; sex: 2 female, 2 males; occurrenceID: 617621A6-E699-5CB3-AAE6-0C461618AD34; **Location:** country: Italy; countryCode: IT; stateProvince: Sicily; municipality: Sortino, Siracusa; locality: Riserva Naturale di Pantalica; **Identification:** identifiedBy: F. Di Giovanni; dateIdentified: 2025; **Event:** eventDate: 05/05/2023; year: 2023; month: 5; day: 5; **Record Level:** institutionCode: FDGC
- **Type status:** Other material. **Occurrence:** recordedBy: S. Cantone, A. Di Giulio; individualCount: 24; sex: 1 females, 23 males; occurrenceID: DD37A48C-6491-5EBB-828F-A0D328756BDE; **Location:** country: Italy; countryCode: IT; stateProvince: Sicily; municipality: Sortino, Siracusa; locality: Riserva Naturale di Pantalica; **Identification:** identifiedBy: F. Di Giovanni; dateIdentified: 2025; **Event:** eventDate: 17/05/2023; year: 2023; month: 5; day: 17; **Record Level:** institutionCode: FDGC
- **Type status:** Other material. **Occurrence:** recordedBy: S. Cantone, A. Di Giulio; individualCount: 2; sex: males; occurrenceID: 6A3109F1-070A-55BA-BB12-370BE9ECC459; **Location:** country: Italy; countryCode: IT; stateProvince: Sicily; municipality: Sortino, Siracusa; locality: Riserva Naturale di Pantalica; **Identification:** identifiedBy: F. Di Giovanni; dateIdentified: 2025; **Event:** eventDate: 17/06/2023; year: 2023; month: 6; day: 17; **Record Level:** institutionCode: FDGC

##### Distribution

Palaearctic (Western Palaearctic).

##### Notes

Species recorded for North and South Italy (Scaramozzino 1995, Pagliano 2009). This is the first record of the subfamily Oxytorinae and the species for Sicily.

#### Phygadeuontinae

Förster, 1869

C18767AF-C05E-595C-96DE-D8A9E31AF340

#### Bathythrix

Förster, 1869

1A9D9488-98CF-5EA3-8A75-0EBB0FDBEBE1

#### Bathythrix lamina

(Thomson, 1884)

96AEF0D3-9AF5-5AE0-8897-10EFF09DB5D0

##### Materials

- **Type status:** Other material. **Occurrence:** recordedBy: S. Cantone, A. Di Giulio; individualCount: 2; sex: females; occurrenceID: 30BD1F35-547C-56BA-B298-A053DFDF2886; **Location:** country: Italy; countryCode: IT; stateProvince: Sicily; municipality: Sortino, Siracusa; locality: Riserva Naturale di Pantalica; **Identification:** identifiedBy: F. Di Giovanni; dateIdentified: 2025; **Event:** eventDate: 27/05/2023; year: 2023; month: 4; day: 2; **Record Level:** institutionCode: FDGC
- **Type status:** Other material. **Occurrence:** recordedBy: S. Cantone, A. Di Giulio; individualCount: 1; sex: male; occurrenceID: 43C9FB59-E4C9-58A9-A2F4-9B4659F0C31A; **Location:** country: Italy; countryCode: IT; stateProvince: Sicily; municipality: Sortino, Siracusa; locality: Riserva Naturale di Pantalica; **Identification:** identifiedBy: F. Di Giovanni; dateIdentified: 2025; **Event:** eventDate: 12/08/2023; year: 2023; month: 8; day: 12; **Record Level:** institutionCode: FDGC

##### Distribution

Palaearctic (Western Palaearctic).

##### Notes

Species recorded for North Italy (Scaramozzino 1995). This is the first record of the species for Sicily.

#### Dichrogaster

Doumerc, 1855

BBE32057-5860-5A0F-9D50-6BD527708277

#### Dichrogaster aestivalis

(Gravenhorst, 1829)

CB24DE56-0737-5BEA-9D9A-8CD2F176CF8D

##### Materials

- **Type status:** Other material. **Occurrence:** recordedBy: S. Cantone, A. Di Giulio; individualCount: 1; sex: male; occurrenceID: A749CC6C-8982-5A5E-955B-D96DEDAECDA0; **Location:** country: Italy; countryCode: IT; stateProvince: Sicily; municipality: Sortino, Siracusa; locality: Riserva Naturale di Pantalica; **Identification:** identifiedBy: F. Di Giovanni; dateIdentified: 2025; **Event:** eventDate: 27/04/2023; year: 2023; month: 04; day: 27; **Record Level:** institutionCode: FDGC
- **Type status:** Other material. **Occurrence:** recordedBy: S. Cantone, A. Di Giulio; individualCount: 2; sex: males; occurrenceID: 5C96B853-605C-5FC9-B0A6-598A04A0DB8A; **Location:** country: Italy; countryCode: IT; stateProvince: Sicily; municipality: Sortino, Siracusa; locality: Riserva Naturale di Pantalica; **Identification:** identifiedBy: F. Di Giovanni; dateIdentified: 2025; **Event:** eventDate: 17/05/2023; year: 2023; month: 05; day: 17; **Record Level:** institutionCode: FDGC
- **Type status:** Other material. **Occurrence:** recordedBy: S. Cantone, A. Di Giulio; individualCount: 2; sex: males; occurrenceID: 5AC367DC-B76C-54C2-92B3-6A1BA6ED05F9; **Location:** country: Italy; countryCode: IT; stateProvince: Sicily; municipality: Sortino, Siracusa; locality: Riserva Naturale di Pantalica; **Identification:** identifiedBy: F. Di Giovanni; dateIdentified: 2025; **Event:** eventDate: 17/06/2023; year: 2023; month: 06; day: 17; **Record Level:** institutionCode: FDGC
- **Type status:** Other material. **Occurrence:** recordedBy: S. Cantone, A. Di Giulio; individualCount: 3; sex: males; occurrenceID: 0BC9C135-3EF5-5F1F-93E8-DF66D24A9AA5; **Location:** country: Italy; countryCode: IT; stateProvince: Sicily; municipality: Sortino, Siracusa; locality: Riserva Naturale di Pantalica; **Identification:** identifiedBy: F. Di Giovanni; dateIdentified: 2025; **Event:** eventDate: 12/08/2023; year: 2023; month: 08; day: 09; **Record Level:** institutionCode: FDGC

##### Distribution

Palaearctic (Eastern Palaearctic; Western Palaearctic).

##### Notes

Species present throughout Italy, including the major islands (Scaramozzino 1995, Generani et al. 2000).

#### Isadelphus

Förster, 1869

AFCDB1ED-592F-55A6-9106-F2604C281FA1

#### Isadelphus armatus

(Gravenhorst, 1829)

FC0F6AD7-BC2F-55EC-A2B8-6A5AF6D0A16B

##### Materials

- **Type status:** Other material. **Occurrence:** recordedBy: S. Cantone, A. Di Giulio; individualCount: 1; sex: male; occurrenceID: A4D33AD3-555E-501C-8800-81E1F04B5DBC; **Location:** country: Italy; countryCode: IT; stateProvince: Sicily; municipality: Sortino, Siracusa; locality: Riserva Naturale di Pantalica; **Identification:** identifiedBy: F. Di Giovanni; dateIdentified: 2025; **Event:** eventDate: 27/04/2023; year: 2023; month: 4; day: 27; **Record Level:** institutionCode: FDGC
- **Type status:** Other material. **Occurrence:** recordedBy: S. Cantone, A. Di Giulio; individualCount: 4; sex: males; occurrenceID: FFCCADF8-C67D-50B9-9F63-5911E0ACA356; **Location:** country: Italy; countryCode: IT; stateProvince: Sicily; municipality: Sortino, Siracusa; locality: Riserva Naturale di Pantalica; **Identification:** identifiedBy: F. Di Giovanni; dateIdentified: 2025; **Event:** eventDate: 17/05/2023; year: 2023; month: 5; day: 17; **Record Level:** institutionCode: FDGC
- **Type status:** Other material. **Occurrence:** recordedBy: S. Cantone, A. Di Giulio; individualCount: 1; sex: female; occurrenceID: 05E45CD6-912E-54D2-8962-133FAC0E3EB5; **Location:** country: Italy; countryCode: IT; stateProvince: Sicily; municipality: Sortino, Siracusa; locality: Riserva Naturale di Pantalica; **Identification:** identifiedBy: F. Di Giovanni; dateIdentified: 2025; **Event:** eventDate: 17/06/2023; year: 2023; month: 6; day: 17; **Record Level:** institutionCode: FDGC

##### Distribution

Palaearctic (Western Palaearctic).

##### Notes

Species recorded for North Italy (Scaramozzino 1995). This is the first record of the species for Sicily.

#### Platyrhabdus

Townes, 1970

AC8F1CFB-81D4-5F6E-BD5F-79F493D7D610

#### Platyrhabdus inflatus

(Thomson, 1884)

CFB03FCE-9F60-57F2-8155-7E48F6B155B8

##### Materials

- **Type status:** Other material. **Occurrence:** recordedBy: S. Cantone, A. Di Giulio; individualCount: 1; sex: female; occurrenceID: F59F4C5D-7AA1-55D9-B1FF-D5188F41ECA2; **Location:** country: Italy; countryCode: IT; stateProvince: Sicily; municipality: Sortino, Siracusa; locality: Riserva Naturale di Pantalica; **Identification:** identifiedBy: F. Di Giovanni; dateIdentified: 2025; **Event:** eventDate: 17/05/2023; year: 2023; month: 5; day: 17; **Record Level:** institutionCode: FDGC

##### Distribution

Palaearctic (Western Palaearctic).

##### Notes

**Figure 18.**
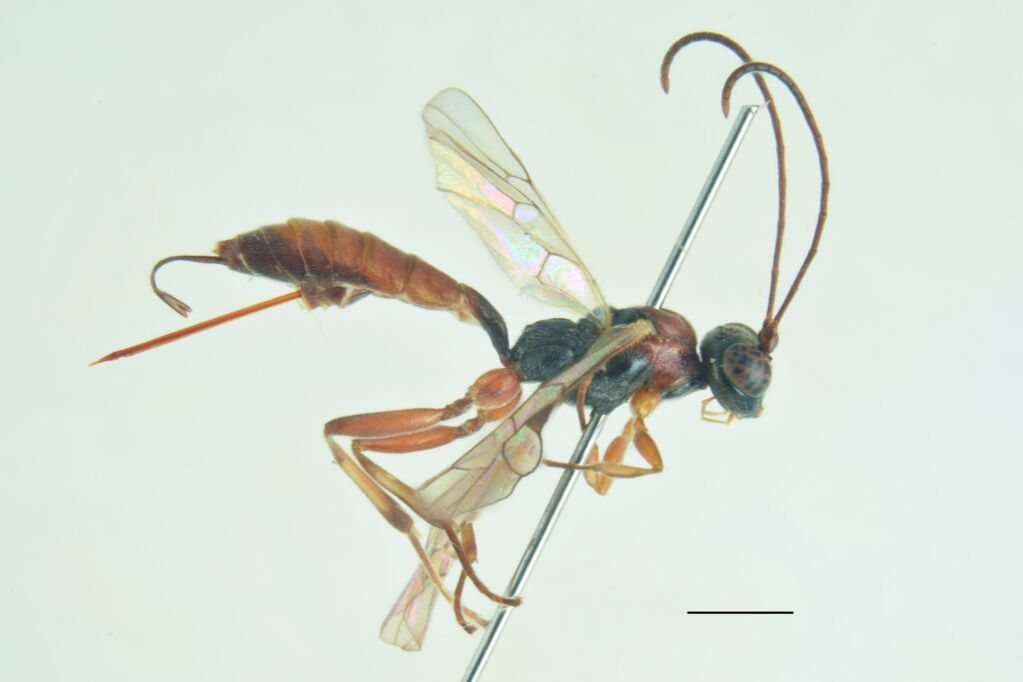
*Platyrhabdus inflatus* (Thomson, 1884), female, habitus. Scale bar 1 mm.

This is the first record of the species for Italy (Fig. 18).

#### Pimplinae

Wesmael, 1845

C7318BF0-60B3-5D18-9855-9BC8D43F9099

#### Delomeristini

Hellén, 1915

34F50BFE-10B9-53AD-BC4D-CF763B3CA51A

#### Perithous

Holmgren, 1859

B10A1CB3-F994-5481-AD92-9FAB3E03AE37

#### Perithous scurra

(Panzer, 1806)

92036DA1-A489-5649-AAC4-2E40B6A78722

##### Materials

- **Type status:** Other material. **Occurrence:** recordedBy: S. Cantone, A. Di Giulio; individualCount: 1; sex: female; occurrenceID: 3FDD928E-2085-515C-8030-541999422DA8; **Location:** country: Italy; countryCode: IT; stateProvince: Sicily; municipality: Sortino, Siracusa; locality: Riserva Naturale di Pantalica; **Identification:** identifiedBy: F. Di Giovanni; dateIdentified: 2025; **Event:** eventDate: 05/05/2023; year: 2023; month: 5; day: 5; **Record Level:** institutionCode: FDGC

##### Distribution

Nearctic; Palaearctic (Eastern Palaearctic; Western Palaearctic).

##### Notes

Species recorded for North and South Italy (Scaramozzino 1995). This is the first record of the species for Sicily.

#### Perithous septemcinctorius

(Thunberg, 1822)

3D616045-27A1-5A2F-91A2-8FFCB63C56E3

##### Materials

- **Type status:** Other material. **Occurrence:** recordedBy: S. Cantone, A. Di Giulio; individualCount: 1; sex: male; occurrenceID: A6E6C007-4F46-5F34-840C-328C2C88353F; **Location:** country: Italy; countryCode: IT; stateProvince: Sicily; municipality: Sortino, Siracusa; locality: Riserva Naturale di Pantalica; **Identification:** identifiedBy: F. Di Giovanni; dateIdentified: 2025; **Event:** eventDate: 27/04/2023; year: 2023; month: 4; day: 27; **Record Level:** institutionCode: FDGC

##### Distribution

Nearctic; Palaearctic (Eastern Palaearctic; Western Palaearctic).

##### Notes

Species recorded for North Italy (Scaramozzino 1995) and Sicily (Zwakhals and Turrisi 2014).

#### Ephialtini

Hellén, 1915

3EFEC538-6A12-5BB2-901D-AB182B779D48

#### Clistopyga

Gravenhorst, 1829

F70A0954-E1A9-58D3-BBD7-6D6860803008

#### Clistopyga incitator

(Fabricius, 1793)

FB55EB87-5AD3-5114-BE40-45AED3DEB21F

##### Materials

- **Type status:** Other material. **Occurrence:** recordedBy: S. Cantone, A. Di Giulio; individualCount: 1; sex: male; occurrenceID: DB274CAE-0F46-5DFD-B668-1154227F1B41; **Location:** country: Italy; countryCode: IT; stateProvince: Sicily; municipality: Sortino, Siracusa; locality: Riserva Naturale di Pantalica; **Identification:** identifiedBy: F. Di Giovanni; dateIdentified: 2025; **Event:** eventDate: 05/05/2023; year: 2023; month: 5; day: 5; **Record Level:** institutionCode: FDGC
- **Type status:** Other material. **Occurrence:** recordedBy: S. Cantone, A. Di Giulio; individualCount: 1; sex: female; occurrenceID: C572E443-9686-5382-8A7F-4A58753D5E04; **Location:** country: Italy; countryCode: IT; stateProvince: Sicily; municipality: Sortino, Siracusa; locality: Riserva Naturale di Pantalica; **Identification:** identifiedBy: F. Di Giovanni; dateIdentified: 2025; **Event:** eventDate: 09/07/2023; year: 2023; month: 7; day: 9; **Record Level:** institutionCode: FDGC

##### Distribution

Afrotropical; Palaearctic (Eastern Palaearctic; Western Palaearctic).

##### Notes

Species present throughout Italy, including the major islands (Scaramozzino 1995, Zwakhals and Turrisi 2014, Korenko and Di Giovanni 2019).

#### Liotryphon

Ashmead, 1900

5C412759-D662-5553-8922-5440CC825852

#### Liotryphon crassiseta

(Thomson, 1877)

1EB9717B-C0AF-5E6C-82C9-C5AA09F98C48

##### Materials

- **Type status:** Other material. **Occurrence:** recordedBy: S. Cantone, A. Di Giulio; individualCount: 1; sex: female; occurrenceID: ADA7D3FA-6E7D-5DE9-B0EC-7D8685CAB70B; **Location:** country: Italy; countryCode: IT; stateProvince: Sicily; municipality: Sortino, Siracusa; locality: Riserva Naturale di Pantalica; **Identification:** identifiedBy: F. Di Giovanni; dateIdentified: 2025; **Event:** eventDate: 27/04/2023; year: 2023; month: 4; day: 27; **Record Level:** institutionCode: FDGC
- **Type status:** Other material. **Occurrence:** recordedBy: S. Cantone, A. Di Giulio; individualCount: 1; sex: female; occurrenceID: 1E184EF6-F7B7-5D8C-A4D9-063C64A3236A; **Location:** country: Italy; countryCode: IT; stateProvince: Sicily; municipality: Sortino, Siracusa; locality: Riserva Naturale di Pantalica; **Identification:** identifiedBy: F. Di Giovanni; dateIdentified: 2025; **Event:** eventDate: 05/05/2023; year: 2023; month: 5; day: 5; **Record Level:** institutionCode: FDGC

##### Distribution

Palaearctic (Eastern Palaearctic; Western Palaearctic).

##### Notes

Species recorded for North and South Italy (Scaramozzino 1995). This is the first record of the species for Sicily.

#### Scambus

Hartig, 1838

D168D7FA-FDA2-5F94-9967-15BB0BF0F975

#### Scambus nigricans

(Thomson, 1877)

A4EB62AA-86E7-5A4D-99A9-5BFBB292EFE9

##### Materials

- **Type status:** Other material. **Occurrence:** recordedBy: S. Cantone, A. Di Giulio; individualCount: 1; sex: female; occurrenceID: 678296C8-DA9C-5F10-9791-51CCD50795D6; **Location:** country: Italy; countryCode: IT; stateProvince: Sicily; municipality: Sortino, Siracusa; locality: Riserva Naturale di Pantalica; **Identification:** identifiedBy: F. Di Giovanni; dateIdentified: 2025; **Event:** eventDate: 17/06/2023; year: 2023; month: 6; day: 17; **Record Level:** institutionCode: FDGC
- **Type status:** Other material. **Occurrence:** recordedBy: S. Cantone, A. Di Giulio; individualCount: 1; sex: male; occurrenceID: AC5BFCD1-7A66-5871-8AE6-EC997C7EBE0D; **Location:** country: Italy; countryCode: IT; stateProvince: Sicily; municipality: Sortino, Siracusa; locality: Riserva Naturale di Pantalica; **Identification:** identifiedBy: F. Di Giovanni; dateIdentified: 2025; **Event:** eventDate: 09/07/2023; year: 2023; month: 7; day: 9; **Record Level:** institutionCode: FDGC

##### Distribution

Palaearctic (Eastern Palaearctic; Western Palaearctic).

##### Notes

Species present throughout Italy, including the major islands (Scaramozzino 1995, Pagliano 2009, Zwakhals and Turrisi 2014).

#### Tromatobia

Förster, 1869

A8C56AD7-FD66-5476-ADF7-6F0447AC40D0

#### Tromatobia ovivora

(Boheman, 1821)

E3DD1DB7-D16D-5FC8-8D66-01911C9C1ED9

##### Materials

- **Type status:** Other material. **Occurrence:** recordedBy: S. Cantone, A. Di Giulio; individualCount: 1; sex: female; occurrenceID: 2C7DBB1C-BE44-569E-8524-BD0C4811447C; **Location:** country: Italy; countryCode: IT; stateProvince: Sicily; municipality: Sortino, Siracusa; locality: Riserva Naturale di Pantalica; **Identification:** identifiedBy: F. Di Giovanni; dateIdentified: 2025; **Event:** eventDate: 05/05/2023; year: 2023; month: 5; day: 5; **Record Level:** institutionCode: FDGC

##### Distribution

Australasian; Nearctic; Neotropical; Palaearctic (Eastern Palaearctic; Western Palaearctic).

##### Notes

Species recorded for North Italy and Sicily (Scaramozzino 1995).

#### Zaglyptus

Förster, 1869

A3F6D9BE-EB09-5F96-A907-94B1C12EA2D6

#### Zaglyptus multicolor

(Gravenhorst, 1829)

622D7A2E-CE80-511E-B7EF-B1D0365BBAFE

##### Materials

- **Type status:** Other material. **Occurrence:** recordedBy: S. Cantone, A. Di Giulio; individualCount: 1; sex: female; occurrenceID: 38968182-5C25-561F-A879-0766F3A4AEB6; **Location:** country: Italy; countryCode: IT; stateProvince: Sicily; municipality: Sortino, Siracusa; locality: Riserva Naturale di Pantalica; **Identification:** identifiedBy: F. Di Giovanni; dateIdentified: 2025; **Event:** eventDate: 27/04/2023; year: 2023; month: 4; day: 27; **Record Level:** institutionCode: FDGC
- **Type status:** Other material. **Occurrence:** recordedBy: S. Cantone, A. Di Giulio; individualCount: 3; sex: females; occurrenceID: C9BFC768-0902-57AB-A6EE-8A67D167D4BF; **Location:** country: Italy; countryCode: IT; stateProvince: Sicily; municipality: Sortino, Siracusa; locality: Riserva Naturale di Pantalica; **Identification:** identifiedBy: F. Di Giovanni; dateIdentified: 2025; **Event:** eventDate: 05/05/2023; year: 2023; month: 5; day: 5; **Record Level:** institutionCode: FDGC
- **Type status:** Other material. **Occurrence:** recordedBy: S. Cantone, A. Di Giulio; individualCount: 1; sex: female; occurrenceID: 9C4C723D-E719-57A5-B755-42C538E0951E; **Location:** country: Italy; countryCode: IT; stateProvince: Sicily; municipality: Sortino, Siracusa; locality: Riserva Naturale di Pantalica; **Identification:** identifiedBy: F. Di Giovanni; dateIdentified: 2025; **Event:** eventDate: 12/08/2023; year: 2023; month: 8; day: 12; **Record Level:** institutionCode: FDGC

##### Distribution

Indomalayan; Palaearctic (Eastern Palaearctic; Western Palaearctic).

##### Notes

Species recorded for North and South Italy (Scaramozzino 1995) and Sicily (De Stefani 1895, Zwakhals and Turrisi 2014, Korenko and Di Giovanni 2019).

#### Pimplini

Wesmael, 1845

BB8BD08D-3CC2-540C-956E-72A5310D8DD8

#### Itoplectis

Förster, 1869

71EE79A3-F191-59CD-B4B9-CD6EA6DC01A2

#### Itoplectis maculator

(Fabricius, 1775)

CBFD4322-E225-5437-A97E-5D7765DD9081

##### Materials

- **Type status:** Other material. **Occurrence:** recordedBy: S. Cantone, A. Di Giulio; individualCount: 3; sex: males; occurrenceID: 70A910B3-F182-5895-BD43-8BB442D92906; **Location:** country: Italy; countryCode: IT; stateProvince: Sicily; municipality: Sortino, Siracusa; locality: Riserva Naturale di Pantalica; **Identification:** identifiedBy: F. Di Giovanni; dateIdentified: 2025; **Event:** eventDate: 05/05/2023; year: 2023; month: 5; day: 5; **Record Level:** institutionCode: FDGC

##### Distribution

Nearctic; Palaearctic (Eastern Palaearctic; Western Palaearctic).

##### Notes

Species present throughout Italy, including the major islands (Scaramozzino 1995).

#### Pimpla

Fabricius, 1804

24BEAF86-E705-52BF-B45A-466FCF6BB3BA

#### Pimpla contemplator

(Müller, 1776)

8D99AB4B-B1DF-5967-B505-16B14ABDA86F

##### Materials

- **Type status:** Other material. **Occurrence:** recordedBy: S. Cantone, A. Di Giulio; individualCount: 1; sex: female; occurrenceID: 160F4E6C-1170-50AB-88A3-69C993DB8450; **Location:** country: Italy; countryCode: IT; stateProvince: Sicily; municipality: Sortino, Siracusa; locality: Riserva Naturale di Pantalica; **Identification:** identifiedBy: F. Di Giovanni; dateIdentified: 2025; **Event:** eventDate: 09/07/2023; year: 2023; month: 7; day: 9; **Record Level:** institutionCode: FDGC

##### Distribution

Palaearctic (Eastern Palaearctic; Western Palaearctic).

##### Notes

Species recorded for North and South Italy (Scaramozzino 1995) and Sicily (Zwakhals and Turrisi 2014).

#### Pimpla rufipes

(Miller, 1759)

E27505B0-2FAC-5E36-AD0D-2660CC795E97

##### Materials

- **Type status:** Other material. **Occurrence:** recordedBy: S. Cantone, A. Di Giulio; individualCount: 1; sex: male; occurrenceID: B2D09203-6F3C-563D-BD00-6D8CE2701616; **Location:** country: Italy; countryCode: IT; stateProvince: Sicily; municipality: Sortino, Siracusa; locality: Riserva Naturale di Pantalica; **Identification:** identifiedBy: F. Di Giovanni; dateIdentified: 2025; **Event:** eventDate: 17/05/2023; year: 2023; month: 5; day: 17; **Record Level:** institutionCode: FDGC
- **Type status:** Other material. **Occurrence:** recordedBy: S. Cantone, A. Di Giulio; individualCount: 1; sex: male; occurrenceID: EE1E56B9-714C-5AC6-86A1-1751B4F65787; **Location:** country: Italy; countryCode: IT; stateProvince: Sicily; municipality: Sortino, Siracusa; locality: Riserva Naturale di Pantalica; **Identification:** identifiedBy: F. Di Giovanni; dateIdentified: 2025; **Event:** eventDate: 17/06/2023; year: 2023; month: 6; day: 17; **Record Level:** institutionCode: FDGC
- **Type status:** Other material. **Occurrence:** recordedBy: S. Cantone, A. Di Giulio; individualCount: 1; sex: female; occurrenceID: 54BD8027-5068-5808-8C7C-6070BD70E778; **Location:** country: Italy; countryCode: IT; stateProvince: Sicily; municipality: Sortino, Siracusa; locality: Riserva Naturale di Pantalica; **Identification:** identifiedBy: F. Di Giovanni; dateIdentified: 2025; **Event:** eventDate: 09/07/2023; year: 2023; month: 7; day: 9; **Record Level:** institutionCode: FDGC

##### Distribution

Australasian; Indomalayan; Palaearctic (Eastern Palaearctic; Western Palaearctic).

##### Notes

Species present throughout Italy, including the major islands (Scaramozzino 1995).

#### Pimpla spuria

Gravenhorst, 1829

CCE65AA3-0ED0-501C-A010-A4F4B8B54157

##### Materials

- **Type status:** Other material. **Occurrence:** recordedBy: S. Cantone, A. Di Giulio; individualCount: 2; sex: 1 female, 1 male; occurrenceID: 5F95C430-0279-5435-B8CA-82643A6274DF; **Location:** country: Italy; countryCode: IT; stateProvince: Sicily; municipality: Sortino, Siracusa; locality: Riserva Naturale di Pantalica; **Identification:** identifiedBy: F. Di Giovanni; dateIdentified: 2025; **Event:** eventDate: 27/04/2023; year: 2023; month: 4; day: 26; **Record Level:** institutionCode: FDGC
- **Type status:** Other material. **Occurrence:** recordedBy: S. Cantone, A. Di Giulio; individualCount: 2; sex: 2 females; occurrenceID: 6BAB8E81-D1E8-5FA9-A0C7-AD200CE6EA37; **Location:** country: Italy; countryCode: IT; stateProvince: Sicily; municipality: Sortino, Siracusa; locality: Riserva Naturale di Pantalica; **Identification:** identifiedBy: F. Di Giovanni; dateIdentified: 2025; **Event:** eventDate: 05/05/2023; year: 2023; month: 5; day: 5; **Record Level:** institutionCode: FDGC
- **Type status:** Other material. **Occurrence:** recordedBy: S. Cantone, A. Di Giulio; individualCount: 3; sex: 1 female, 2 males; occurrenceID: BC3B0255-8ECA-5F0C-954E-6925E8F0717F; **Location:** country: Italy; countryCode: IT; stateProvince: Sicily; municipality: Sortino, Siracusa; locality: Riserva Naturale di Pantalica; **Identification:** identifiedBy: F. Di Giovanni; dateIdentified: 2025; **Event:** eventDate: 17/05/2023; year: 2023; month: 5; day: 17; **Record Level:** institutionCode: FDGC

##### Distribution

Indomalayan; Palaearctic (Eastern Palaearctic; Western Palaearctic).

##### Notes

Species recorded for North, South Italy and Sicily (Scaramozzino 1995).

#### Poemeniinae

Narayanan & Lal, 1953

3F4D41E2-F8D4-5386-A57B-B64D42B403BB

#### Podoschistus

Townes, 1957

BC0A8663-D01F-557C-B6F8-57D3573BC768

#### Podoschistus scutellaris

(Desvignes, 1856)

66FA3F04-29B5-5838-940B-EF96ECD7F1CF

##### Materials

- **Type status:** Other material. **Occurrence:** recordedBy: S. Cantone, A. Di Giulio; individualCount: 1; sex: female; occurrenceID: 9A20249B-2C96-5380-8FAD-9104FDB0E7AB; **Location:** country: Italy; countryCode: IT; stateProvince: Sicily; municipality: Sortino, Siracusa; locality: Riserva Naturale di Pantalica; **Identification:** identifiedBy: F. Di Giovanni; dateIdentified: 2025; **Event:** eventDate: 09/07/2023; year: 2023; month: 7; day: 9; **Record Level:** institutionCode: FDGC

##### Distribution

Indomalayan; Palaearctic (Western Palaearctic).

##### Notes

Species recorded for North Italy (Pagliano 2009, Di Giovanni et al. 2015) and Sardinia (Di Giovanni and Reshchikov 2016). This is the first record of the species for Sicily.

#### Stilbopinae

Townes & Townes, 1949

D11FFDC0-1725-52DA-B3C3-F71B7EEDAF97

#### Stilbops

Förster, 1869

658E53D6-C08A-5ECE-83D0-0A545B10FA1C

#### Stilbops (Stilbops) vetulus

(Gravenhorst, 1829)

70D7EC70-9422-5C62-BD95-4954A21100E1

##### Materials

- **Type status:** Other material. **Occurrence:** recordedBy: S. Cantone, A. Di Giulio; individualCount: 1; sex: female; occurrenceID: 8DC24893-5CE0-54BD-940F-8CFB7D414964; **Location:** country: Italy; countryCode: IT; stateProvince: Sicily; municipality: Sortino, Siracusa; locality: Riserva Naturale di Pantalica; **Identification:** identifiedBy: F. Di Giovanni; dateIdentified: 2025; **Event:** eventDate: 27/04/2023; year: 2023; month: 4; day: 27; **Record Level:** institutionCode: FDGC

##### Distribution

Palaearctic (Eastern Palaearctic; Western Palaearctic).

##### Notes

Species recorded for North and South Italy (Scaramozzino 1995, Corcos et al. 2017). This is the first record of the species for Sicily.

#### Tryphoninae

Shuckard, 1840

7A2BD022-8D2D-5626-8241-FFB1DFBF9E82

#### Oedemopsini

Woldstedt, 1877

1B0595B1-236F-5928-974C-657E86F53797

#### Thymaris

Förster, 1869

21188538-0EFC-5606-9861-49BD6EDD65C6

#### Thymaris niger

(Taschenberg, 1865)

EB0E5350-FC20-5A55-9DE2-AE8E37C2A79F

##### Materials

- **Type status:** Other material. **Occurrence:** recordedBy: S. Cantone, A. Di Giulio; individualCount: 1; sex: male; occurrenceID: F47EEB85-9C0D-5007-B0C4-10B314F3EFEB; **Location:** country: Italy; countryCode: IT; stateProvince: Sicily; municipality: Sortino, Siracusa; locality: Riserva Naturale di Pantalica; **Identification:** identifiedBy: F. Di Giovanni & G. Broad; dateIdentified: 2025; **Event:** eventDate: 17/06/2023; year: 2023; month: 6; day: 17; **Record Level:** institutionCode: FDGC

##### Distribution

Palaearctic (Eastern Palaearctic; Western Palaearctic).

##### Notes

Species recorded for North and South Italy (Scaramozzino 1995). This is the first record of the species for Sicily.

#### " Exenterini "

Förster, 1869

2B30A119-8B7D-503F-AD3E-607A37F3AD0E

#### Acrotomus

Holmgren, 1857

8A76473A-484C-5685-ADA1-F958A2832F39

#### Acrotomus lucidulus

(Gravenhorst, 1829)

C12BB82E-1F34-5C22-B398-4D711F952AA7

##### Materials

- **Type status:** Other material. **Occurrence:** recordedBy: S. Cantone, A. Di Giulio; individualCount: 2; sex: males; occurrenceID: 8F3EE31E-AA27-596C-B7A0-2BB6328847CC; **Location:** country: Italy; countryCode: IT; stateProvince: Sicily; municipality: Sortino, Siracusa; locality: Riserva Naturale di Pantalica; **Identification:** identifiedBy: F. Di Giovanni; dateIdentified: 2025; **Event:** eventDate: 27/04/2023; year: 2023; month: 4; day: 27; **Record Level:** institutionCode: FDGC
- **Type status:** Other material. **Occurrence:** recordedBy: S. Cantone, A. Di Giulio; individualCount: 1; sex: male; occurrenceID: F25684B5-471B-54A2-A77A-CEE747241430; **Location:** country: Italy; countryCode: IT; stateProvince: Sicily; municipality: Sortino, Siracusa; locality: Riserva Naturale di Pantalica; **Identification:** identifiedBy: F. Di Giovanni; dateIdentified: 2025; **Event:** eventDate: 05/05/2023; year: 2023; month: 5; day: 5; **Record Level:** institutionCode: FDGC

##### Distribution

Palaearctic (Eastern Palaearctic; Western Palaearctic).

##### Notes

Species recorded for North and South Italy (Scaramozzino 1995, Pagliano 2009). This is the first record of the species for Sicily.

#### Cycasis

Townes, 1965

EF585EF6-3896-593E-8B12-FA1E1F27E149

#### Cycasis rubiginosa

(Gravenhorst, 1829)

B9D0F8AF-350C-549C-BCBF-898A7DE8BB63

##### Materials

- **Type status:** Other material. **Occurrence:** recordedBy: S. Cantone, A. Di Giulio; individualCount: 1; sex: male; occurrenceID: 758B23B5-61A8-5EE9-847C-3134278CBC59; **Location:** country: Italy; countryCode: IT; stateProvince: Sicily; municipality: Sortino, Siracusa; locality: Riserva Naturale di Pantalica; **Identification:** identifiedBy: F. Di Giovanni; dateIdentified: 2025; **Event:** eventDate: 27/04/2023; year: 2023; month: 4; day: 27; **Record Level:** institutionCode: FDGC
- **Type status:** Other material. **Occurrence:** recordedBy: S. Cantone, A. Di Giulio; individualCount: 1; sex: female; occurrenceID: 9A005770-3337-5AFF-AA78-87B41C2E5F23; **Location:** country: Italy; countryCode: IT; stateProvince: Sicily; municipality: Sortino, Siracusa; locality: Riserva Naturale di Pantalica; **Identification:** identifiedBy: F. Di Giovanni; dateIdentified: 2025; **Event:** eventDate: 05/05/2023; year: 2023; month: 5; day: 5; **Record Level:** institutionCode: FDGC

##### Distribution

Palaearctic (Eastern Palaearctic; Western Palaearctic).

##### Notes

Species originally described from South Italy (Tuscany) (Gravenhorst 1829), recorded also from North Italy (Scaramozzino 1995). This is the first record of the species for Sicily.

#### Tryphonini

Shuckard, 1840

40CD0C76-7F8F-5DA9-BDF1-D5471063FC44

#### Ctenochira

Förster, 1855

178138D3-A3F2-5DBC-A84D-4415BA45C92D

#### Ctenochira genalis

(Thomson, 1883)

23B04090-6810-520A-87FD-C0710FDB458B

##### Materials

- **Type status:** Other material. **Occurrence:** recordedBy: S. Cantone, A. Di Giulio; individualCount: 2; sex: 1 female, 1 male; occurrenceID: EB217F8B-10F4-5AC4-9E85-E01DC80BFF04; **Location:** country: Italy; countryCode: IT; stateProvince: Sicily; municipality: Sortino, Siracusa; locality: Riserva Naturale di Pantalica; **Identification:** identifiedBy: F. Di Giovanni; dateIdentified: 2025; **Event:** eventDate: 27/04/2023; year: 2023; month: 4; day: 27; **Record Level:** institutionCode: FDGC

##### Distribution

Palaearctic (Eastern Palaearctic; Western Palaearctic).

##### Notes

Species recorded for North and South Italy (Di Giovanni and Reshchikov 2016). This is the first record of the species for Sicily.

#### Xoridinae

Shuckard, 1840

2905697B-1F21-50C2-B154-3334EF0B9A41

#### Xorides

Latreille, 1809

16569CD1-EDFA-5524-8151-118A65CF3F91

#### Xorides praecatorius

(Fabricius, 1793)

231FC804-9BD6-5031-87F2-1B302D76FC62

##### Materials

- **Type status:** Other material. **Occurrence:** recordedBy: S. Cantone, A. Di Giulio; individualCount: 1; sex: femal; occurrenceID: 5FF39678-A7A7-5F04-9C9E-6C8E3BFA3862; **Location:** country: Italy; countryCode: IT; stateProvince: Sicily; municipality: Sortino, Siracusa; locality: Riserva Naturale di Pantalica; **Identification:** identifiedBy: F. Di Giovanni; dateIdentified: 2025; **Event:** eventDate: 17/06/2023; year: 2023; month: 6; day: 17; **Record Level:** institutionCode: FDGC

##### Distribution

Palaearctic (Eastern Palaearctic; Western Palaearctic)

##### Notes

Species recorded for North and South Italy (Scaramozzino 1995). This is the first record of the species for Sicily.

## Discussion

Based on the data from the previous checklist of Ichneumonidae (Scaramozzino 1995), Sicily appears to be one of the Italian regions with the highest diversity, ranking after northern regions, such as Piedmont, Trentino-Alto Adige and Emilia-Romagna (Di Giovanni & Dal Pos, unpublished data). This is partly a result of the checklist of the Italian fauna treating Sicily and Sardinia as distinct biogeographic units (Minelli et al. 1995).

However, this paper reveals that significant gaps remain in our understanding of the distribution of these insects on the island. Most of the available data originate from very old and outdated sources, such as De Stefani (1895) and for some subfamilies — for example, Ctenopelmatinae, Mesochorinae, Orthocentrinae — the data included in the 1995 checklist are essentially absent. Conversely, the groups for which information is more complete are those that have received renewed attention in recent years, such as Diplazontinae, Ichneumoninae and Pimplinae (Turrisi et al. 2007, Riedel and Turrisi 2013, Zwakhals and Turrisi 2014).

These findings highlight how significantly the true diversity of this group of parasitoid Hymenoptera in Italy remains underestimated and underscore the need to fill current gaps in faunistic knowledge of several animal taxa in the country, particularly those with a Mediterranean-centred distribution.

## Figures and Tables

**Table 1.** P-distance values obtained by comparing the newly-deposited *M. obscurus* sequence (PX272273) with the two best hits retrieved from BOLD and NCBI databases.

		P-distance (%)
*M. oculatus* (GMFIE519-12)	*M. oculatus* (MZ607881)	0.34
*M. oculatus* (GMFIE519-12)	*M. obscurus* (PX272273, this study)	5.41
*M. oculatus* (MZ607881)	*M. obscurus* (PX272273, this study)	5.84
